# Hypoxia enhances the malignant nature of bladder cancer cells and concomitantly antagonizes protein *O*-glycosylation extension

**DOI:** 10.18632/oncotarget.11257

**Published:** 2016-08-12

**Authors:** Andreia Peixoto, Elisabete Fernandes, Cristiana Gaiteiro, Luís Lima, Rita Azevedo, Janine Soares, Sofia Cotton, Beatriz Parreira, Manuel Neves, Teresina Amaro, Ana Tavares, Filipe Teixeira, Carlos Palmeira, Maria Rangel, André M.N. Silva, Celso A. Reis, Lúcio Lara Santos, Maria José Oliveira, José Alexandre Ferreira

**Affiliations:** ^1^ Experimental Pathology and Therapeutics Group, IPO Porto Research Center (CI-IPOP), Portuguese Oncology Institute of Porto (IPO Porto), Porto, Portugal; ^2^ New Therapies Group, INEB-Institute for Biomedical Engineering, Porto, Portugal; ^3^ Instituto de Investigação e Inovação em Saúde, Universidade do Porto, Portugal; ^4^ Institute of Biomedical Sciences Abel Salazar, University of Porto, Porto, Portugal; ^5^ Biomaterials for Multistage Drug and Cell Delivery, INEB-Institute for Biomedical Engineering, Porto, Portugal; ^6^ Glycobiology in Cancer, Institute of Molecular Pathology and Immunology of the University of Porto (IPATIMUP), Porto, Portugal; ^7^ Department of Pathology, Hospital Pedro Hispano, Matosinhos, Portugal; ^8^ LAQV-REQUIMTE, Faculty of Sciences of the University of Porto, Porto, Portugal; ^9^ Health School of University Fernando Pessoa, Porto, Portugal; ^10^ UCIBIO-REQUIMTE, Instituto de Ciências Biomédicas Abel Salazar, University of Porto, Porto, Portugal; ^11^ UCIBIO-REQUIMTE/Department of Chemistry and Biochemistry, Faculty of Sciences, University of Porto, Porto, Portugal; ^12^ Department of Pathology and Oncology, Faculty of Medicine, Porto University, Porto, Portugal; ^13^ Department of Surgical Oncology, Portuguese Institute of Oncology, Porto, Portugal; ^14^ Porto Comprehensive Cancer Center (P.ccc), Porto, Portugal

**Keywords:** glycosylation, bladder cancer, hypoxia, invasion, sialyl-Tn

## Abstract

Invasive bladder tumours express the cell-surface Sialyl-Tn (STn) antigen, which stems from a premature stop in protein O-glycosylation. The STn antigen favours invasion, immune escape, and possibly chemotherapy resistance, making it attractive for target therapeutics. However, the events leading to such deregulation in protein glycosylation are mostly unknown. Since hypoxia is a salient feature of advanced stage tumours, we searched into how it influences bladder cancer cells glycophenotype, with emphasis on STn expression. Therefore, three bladder cancer cell lines with distinct genetic and molecular backgrounds (T24, 5637 and HT1376) were submitted to hypoxia. To disclose HIF-1α-mediated events, experiments were also conducted in the presence of Deferoxamine Mesilate (Dfx), an inhibitor of HIF-1α proteasomal degradation. In both conditions all cell lines overexpressed HIF-1α and its transcriptionally-regulated protein CA-IX. This was accompanied by increased lactate biosynthesis, denoting a shift toward anaerobic metabolism. Concomitantly, T24 and 5637 cells acquired a more motile phenotype, consistent with their more mesenchymal characteristics. Moreover, hypoxia promoted STn antigen overexpression in all cell lines and enhanced the migration and invasion of those presenting more mesenchymal characteristics, in an HIF-1α-dependent manner. These effects were reversed by reoxygenation, demonstrating that oxygen affects *O*-glycan extension. Glycoproteomics studies highlighted that STn was mainly present in integrins and cadherins, suggesting a possible role for this glycan in adhesion, cell motility and invasion. The association between HIF-1α and STn overexpressions and tumour invasion was further confirmed in bladder cancer patient samples. In conclusion, STn overexpression may, in part, result from a HIF-1α mediated cell-survival strategy to adapt to the hypoxic challenge, favouring cell invasion. In addition, targeting STn-expressing glycoproteins may offer potential to treat tumour hypoxic niches harbouring more malignant cells.

## INTRODUCTION

Muscle invasive bladder cancer (MIBC) is the second deadliest genitourinary cancer [1]. The mainstay treatment includes surgery and (neo)adjuvant chemotherapy. However, due to treatment failure, the five-year overall survival does not exceed 50%, urging the introduction of novel biomarkers to aid patient stratification and effective target therapeutics [2].

Growth under oxygen deficiency (hypoxia) and changes in the glycosylation of cell-surface proteins are salient features of solid tumors, which often correlate with advanced stages of malignancy. The primary factor mediating cellular responses to hypoxia is the hypoxia-inducible factor-1α (HIF-1α), which transcriptional activity is oxygen-dependent [3, 4]. The HIF-1α protein is constantly synthesized in the cytosol, escaping, under low oxygen pressure, proteasomal degradation [3, 4]. Once translocated to the nucleus, HIF-1α activates an array of adaptive responses that include overexpression of angiogenic, anti-apoptotic and growth factor genes, as well as a shift from aerobic to anaerobic metabolism [3, 4]. Hypoxic niches also contribute to the transformation and survival of chemoresistant cancer stem cells capable of recapitulating tumour heterogeneity and, ultimately, invade and disseminate via the acquisition of mesenchymal traits [5, 6]. Therefore, the design of targeted therapies against hypoxic cells is of major importance to disease management.

Glycosylation alterations are frequently observed in cancer and have been shown to play key roles in tumour progression [7]. Few studies have demonstrated that hypoxia and HIF-1α can interfere in the modulation of cell-surface glycosylation by promoting an unbalanced expression of glycosyltransferases, sugar transporters, and nucleotide sugars biosynthesis enzymes [8–10]. By influencing glycosylation, hypoxia indirectly modulates cell survival signalling pathways, cell-cell adhesion, invasion and immune recognition, among other vital biological functions [10–12]. Nevertheless, the interdependence between hypoxia and glycosylation-driven events, as well as the potential of hypoxia-associated glycans for guided therapeutics are still poorly understood.

**Figure 1:**
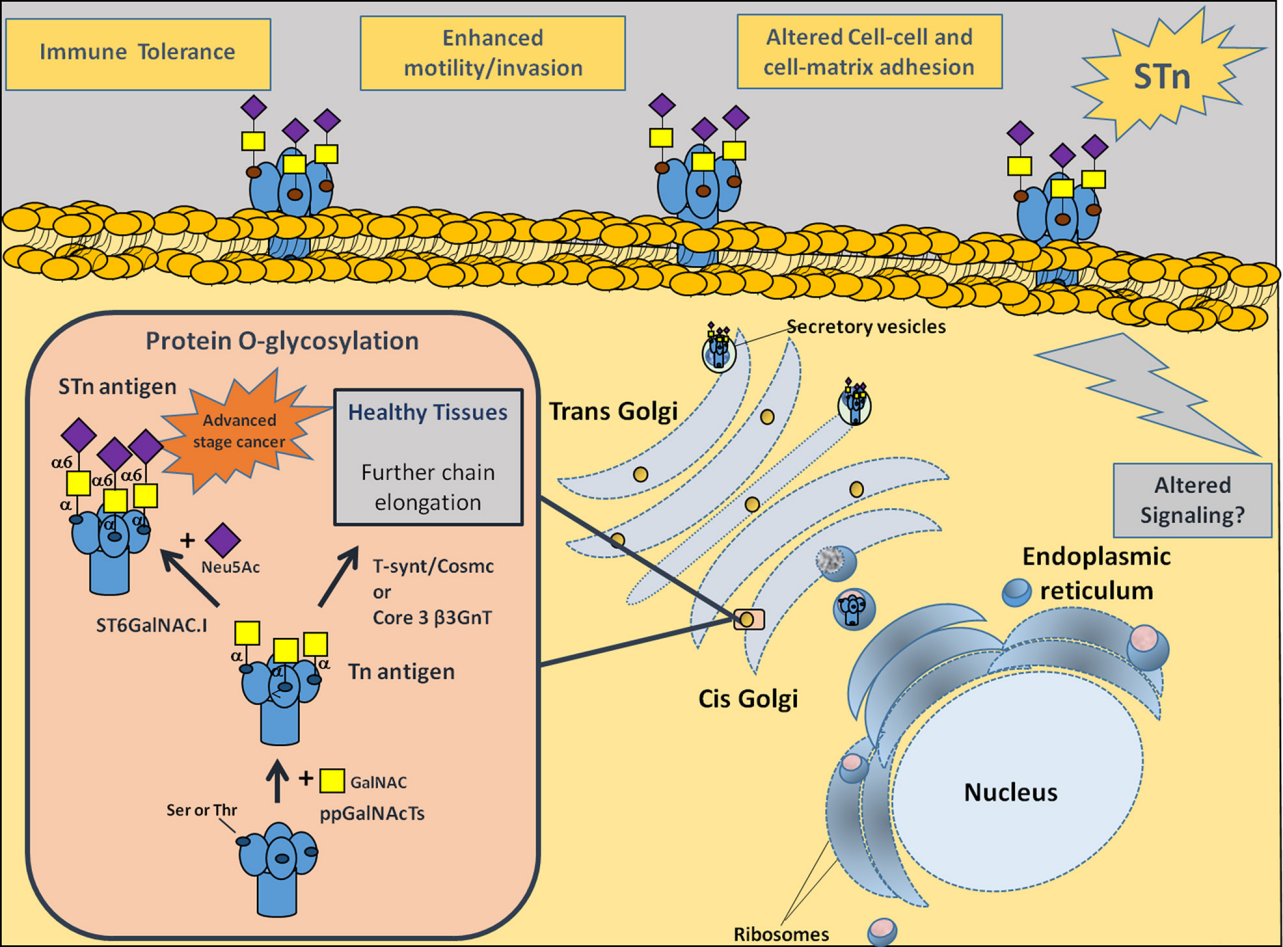
Representation of membrane proteins O-glycosylation (Ser/Thr residues) in advanced stage bladder tumours, with emphasis on STn biosynthesis Protein glycosylation is a highly regulated process of critical importance for protein stability and function. Newly synthesized proteins are O-glycosylated in the Golgi apparatus by ppGalNAcTs. These enzymes are responsible by the addition of a GalNAc moiety to Ser/Thr residues, originating the Tn antigen (GalNAc-O-Ser/Thr-protein backbone), the simplest O-glycan. In healthy and/or less malignant cells, O-glycans are extended through the sequential addition of other sugars first by C1GalT1 and its chaperone Cosmc and then other glycosyltransferases. This culminates in highly complex, heterogeneous and elongated glycans often terminated by ABO or Lewis blood group related antigens (not represented). In cancer cells, the Tn antigen is immediately sialylated by ST6GalNAc-I, originating the STn antigen (Neu5Ac-GalNAc-O-Ser/Thr-protein backbone), thereby inhibiting further chain elongation. The expression of STn at the cell surface influences cell-cell adhesion and cancer cell recognition, favouring motility, invasion, immune escape and possibly altering intracellular signalling.

Recently we described that the majority of muscle-invasive bladder cancers (MIBC), which are highly hypoxic [13, 14], mimic other advanced stage solid tumours [15–19] by overexpressing the cancer-associated carbohydrate antigen sialyl-Tn (STn) [20, 21]. This antigen usually stems from a premature stop in *O*-glycosylation (linked to Ser/Thr) of cell-surface proteins due to ST6GalNAc-I activity [22] (Figure 1), and it is not expressed in healthy urothelium. In tumours, it is markedly expressed in non-proliferative areas known for their resistance to chemotherapy [23]. Furthermore, we found that STn expression modulated cell-surface glycoproteins function, favouring cancer cell migration and invasion [21, 24], as well as induced immune tolerance [20] (Figure 1). Nevertheless, the biological events triggering STn overexpression in human cancers remain to be fully understood. Building on these insights and envisaging the development of a strategy to target hypoxic cancer cells, we comprehensively address the influence of hypoxia in bladder cancer aggressiveness and, for the first time, in STn overexpression.

## RESULTS AND DISCUSSION

Hypoxia and altered glycosylation are salient features of advanced stage bladder cancer, with negative implications in the disease outcome. However, the modulation of cell glycosylation by hypoxia and its biological significance is mostly an unexplored matter. This work addresses how hypoxia alters the glycophenotype of bladder cancer cells, with emphasis on changes in protein O-glycosylation characterized by the overexpression of the STn antigen.

### Hypoxia driven molecular and morphological changes of bladder cancer cells

The first part of the study was devoted to evaluating the expression of the hypoxia biomarker HIF-1α and its transcriptionally-regulated protein CA-IX in three bladder cancer cell lines with distinct genetic and molecular backgrounds (T24, 5637, HT1376) under hypoxia. The cells were primarily exposed to hypoxia (0.1% O_2_) for 6, 12 and 24 h envisaging the optimization of the hypoxia exposure time for further analysis. Experiments were also conducted in normoxia and in the presence of Dfx, which inhibits Prolyl Hydroxylases by chelating Fe^2+^ [25, 26], thereby inhibiting HIF-1α proteasomal degradation [26].

**Figure 2:**
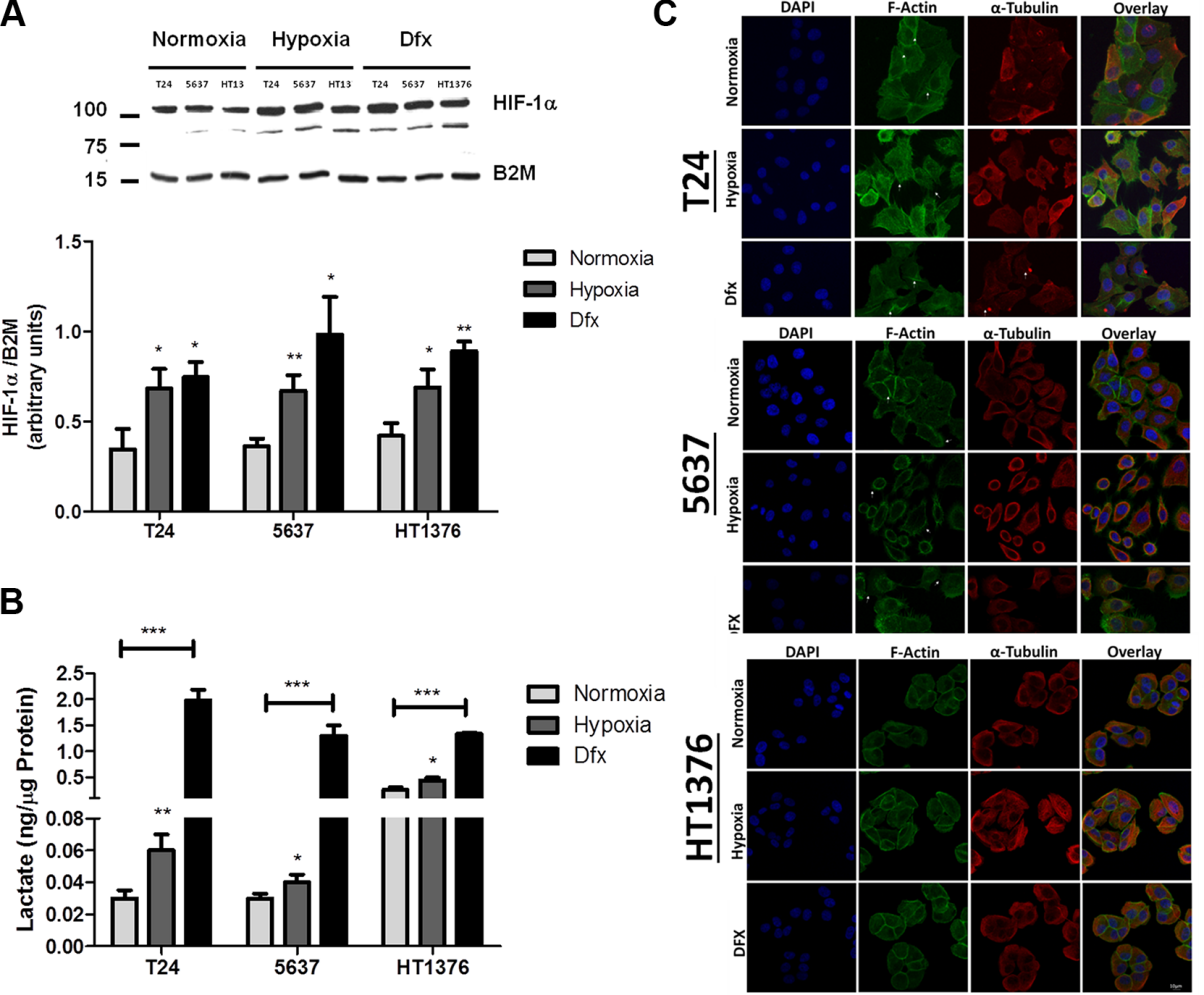
Molecular and morphological changes induced by hypoxia in bladder cancer cell lines (**A**) Variations in HIF-1α in bladder cancer cells (T24, 5637, HT1376) exposed to hypoxia (0.1% O_2_; 24 h for T24 and 5637 and 6 h for HT1376) and Dfx in relation to normoxia (21.0% O_2_). All cell lines significantly increased the levels of HIF-1α in hypoxia and Dfx when compared to normoxia. (**B**) Lactate production in bladder cancer cells under normoxia, hypoxia and Dfx exposure. Growth under hypoxia or in the presence of Dfx promoted a significant increase in lactate production, which was more pronounced for Dfx conditions. These observations denote a shift towards anaerobic metabolism. (**C**) Effect of hypoxia on bladder cancer cell morphology and actin/tubulin cytoskeleton organization. F-actin and tubulin staining was performed in PFA fixed cells after exposure to normoxic, hypoxic and DFX conditions for 24 h (T24 and 5637) or 6 h (HT1376). F-actin was stained with Phalloidin-FITC (green), while α-tubulin was stained with a monoclonal antibody following incubation with an AlexaFluor594 secondary antibody (red). In turn, nuclei were counterstained with DAPI (blue). Scale bar represents 10 μm. Normoxic T24 cells form aggregated islands with evident actin staining at cell-cell adhesion sites (arrowheads). Membranes of outer cells present reduced projections and some actin stress fibbers are visible (arrows). Under hypoxic conditions, these cells present dissociated and scattered cell aggregates with pronounced and abundant actin filopodia (arrows) present at cell periphery. In comparison to normoxic conditions, less stress fibbers are visible. When exposed to DFX, T24 cells present an intermediate cell morphology between normoxia and hypoxia. Cell aggregates are not as cohesive as in normoxia and not as scattered as in hypoxia. Cells show reduced cell filopodia and exhibit abundant stress fibbers (arrows) co-localized with α-tubulin-rich focal adhesions (arrows). Under normoxia, 5637 cells present loosed islands with fewer cell-cell contact points (arrows) than the other cell lines. Short filopodia is seen at the cell periphery (arrows). In hypoxic conditions, 5637 cells appear as isolated cells with heterogeneous morphology. Pronounced, abundant and long actin filopodia (arrows) is present at cell periphery and no stress fibbers are visible. DFX exposure of these cells results in an intermediate cell morphology between normoxia and hypoxia. Cell islands seem to disperse and adhesion contacts are mainly established through cellular projections (arrows). Cell periphery exhibits lamellipodia and long filopodia. In both normoxic and hypoxic conditions, HT1376 cells form small cell islands, establishing cell contact through minor cell projections. Cell-cell adhesion areas are less enriched in actin filaments and no stress fibbers are visible. Under DFX exposure, cell islands are more cohesive with intense actin staining at cell-cell adhesion contacts. Cell periphery does not present filopodia nor lamellipodia. In resume, exposure to hypoxia drives cells to accumulate hypoxia marker HIF-1α, shift towards an anaerobic metabolism and to significant morphological alterations towards a less cohesive cell phenotype. These alterations are significantly more pronounced under Dfx exposure, highlighting the role of HIF-1α in the establishment of molecular alterations associated with hypoxia. Graphs represent average value of three independent experiments, *p < 0.05; **p < 0.01; ***p < 0.001 (Student's *T*-test).

Western blots for HIF-1α showed a more intense band at 110 kDa and an additional band at 92 kDa (Figure 2A), which has been suggested to be a splice variant of this protein [27]. Nevertheless, both bands showed similar variations between conditions and were taken into consideration in the quantitative evaluation of HIF-1α expression. Accordingly, HIF-1α was significantly increased in T24 and 5637 cells after 24 h of hypoxia exposure in relation to normoxia. For HT1376 cells this was an earlier event initiated after 6 h of hypoxia induction. Similar results were observed for cells grown in the presence of Dfx, confirming the stabilization of HIF-1α. The CA-IX protein followed the HIF-1α expression tendency both under hypoxia and Dfx exposure (Supplementary Figure S1), demonstrating an active HIF-1α regulation of gene expression. Based on these observations, subsequent studies with T24 and 5637 were conducted at 24 h whereas studies involving HT1376 were performed at 6 h time exposures. The increase in HIF-1α already at 6 h for HT1376 may be related with its intrinsic deletion of PTEN and inactivation of p53 genes, which have been described to potentiate HIF-1α transcription [28].

The overexpression of hypoxia markers was accompanied by an increase in lactate levels in the culture media for both hypoxia and Dfx, demonstrating a shift towards anaerobic metabolism characteristic of hypoxic conditions (Figure 2B). A significant decrease in cell proliferation is also observed under hypoxia and Dfx (Supplementary Figure S2), which is consistent with previous reports regarded with hypoxia and HIF-1α mediated events [29, 30]. Moreover, HT1376 showed a 10-fold lower proliferation rate when compared to the other two cell lines irrespectively of the conditions, highlighting a marked differentiated character. In agreement with these findings, we have previously reported that HT1376 cell line is more resistant than T24 and 5637 to cisplatin, the main anti-proliferative agents used in clinical settings for bladder cancer [31]. Despite these observations no significant alterations in cell viability were observed in any of the studied conditions (Supplementary Figure S3).

In addition to molecular and metabolic alterations, cell lines dramatically changed their morphology under hypoxia and Dfx (Figure 2C). Under normoxia, T24 cells presented mixed epithelioid-fibroblastoid morphology; however, under hypoxia and Dfx conditions, cells acquired a more elongated semblance and presented larger intercellular spaces, suggesting an HIF-1α mediated phenomenon. In agreement with these observations, F-actin and α-tubulin stainings evidenced that hypoxic T24 cells presented dissociated and scattered cell aggregates with pronounced and abundant actin filopodia at cell periphery. In comparison to normoxic conditions, less stress fibbers were observed in hypoxic cells. Dfx-exposed T24 cells were not as cohesive as in normoxia and not as scattered as in hypoxia, highlighting an intermediate phenotype between both conditions. Interestingly, α-tubulin and F-actin staining also revealed focal contacts that are localized at the tip of actin stress fibbers. These contractile actomyosin bundles anchored to focal adhesions connect the extracellular matrix (ECM) to the actin cytoskeleton and convert mechanical signals into biochemical cues, having an important role in focal adhesion maturation and dynamics [32]. Such crosstalk was also visible in Dfx conditions, suggesting the involvement of HIF-1α in these processes. On their turn, under normoxia, 5637 cells presented polyhedral morphology with peripheral membrane ruffling. Concomitantly, F-actin and α-tubulin stainings showed small islands with established cell-cell contact points with evident cortical actin staining and short filopodia the cell periphery. Under hypoxia these cells became scattered and morphologically heterogeneous. Pronounced, abundant and long actin filopodia were observed at cell periphery and no stress fibbers were visible. Again, exposure to Dfx translated into intermediate cell morphology between normoxia and hypoxia. However, cell islands seemed to disperse and adhesion contacts were mainly established through cellular projections. The cell periphery exhibited lamellipodia and long filopodia. In clear contrast with the other cell lines, normoxic HT1376 cells formed small cell islands, establishing cell contacts through minor cellular projections. Cell-cell adhesion areas were less enriched in actin filaments and no stress fibbers were visible. Cells grown under hypoxia and Dfx retained a similar morphology and did not show major alterations in cytoskeleton dynamics, even though some areas suggested increased cell scattering. However, under Dfx exposure, cell islands were more cohesive with intense actin staining at cell-cell adhesion contacts. Cell periphery did not present filopodia or lamellipodia in hypoxia and Dfx, again in opposition to T24 and 5637 cells. The acquisition of these two major types of actin-based protrusions by T24 and 5637 but not by HT1376 cells under hypoxia, suggest the establishment of phenotypical features related with increased cell motility.

In summary, both hypoxia and exposure to HIF-1α inhibitor Dfx led to the activation of hypoxia-related cellular responses translated by an increase in HIF-1α and CA-IX expression, a shift towards anaerobic metabolism and significant alterations in cell morphology and cytoskeleton dynamics towards a less cohesive/motile phenotype.

### Hypoxic regulation of gene expression

Bladder cancer cells were further characterized in relation to the expression of a panel of 21 genes associated with stem, epithelial, epithelial-to-mesenchymal transition, and mesenchymal cell phenotypes. Based on the heat map in Supplementary Figure S4A, our results evidenced that under normoxia T24 and 5637 cells presented lower expression of epithelial markers (*CDH1, EPCAM* and *DSP*) accompanied by an upregulation of mesenchymal-characteristic genes (*CDH2*, *FN1*, *SPARC*, and *VIM*) when compared to HT1376. The classical mesenchymal phenotype was particularly evident in the 5637 cell line whereas the T24 cell line showed more pronounced stem cell characteristics, translated by the overexpression of *NANOG*, *LIN28* and, *SOX2*. Again contrasting with the other two cell lines, HT1376 cells showed higher expression of epithelial markers while downregulating mesenchymal associated genes. In spite of HT1376 cells are genetically related to 5637 [33], 5637 cell line is more similar to T24 cells at the transcriptomic level. Exposure to hypoxia promoted significant alterations in the gene expression patterns of T24 and particularly of 5637 cells; conversely little alterations were found in HT1376 under hypoxia (Supplementary Figure S4B). These results reinforced the similarities between T24 and 5637 cells observed at a morphological level. The integrative analysis of T24 gene expression profiles using ClueGo and CluePedia plugins for Cytoscape further highlighted a positive regulation of stem cell proliferation, reinforcing the stem cell character of these cells, accompanied by the negative regulation of cell adhesion under hypoxia (Supplementary Figure S4C), thus in agreement with the morphological analysis. The comprehensive genetic analysis of 5637 cells highlighted possible deregulations in stem cell proliferation as well as in the activation of the Wnt signalling pathway, which has been associated with the invasive and metastatic potential of cells [34, 35]. The few alterations presented by HT1376 under hypoxia did not allow this type of analysis. In summary, our data suggests that T24 and 5637 bladder cancer cells, present a more pronounced stem and mesenchymal character, are more extensively affected by hypoxia. In particular, these cell were endowed with a more motile phenotype compared to HT1376, which showed a more marked epithelial nature.

### Hypoxic regulation of glycosylation

**Figure 3:**
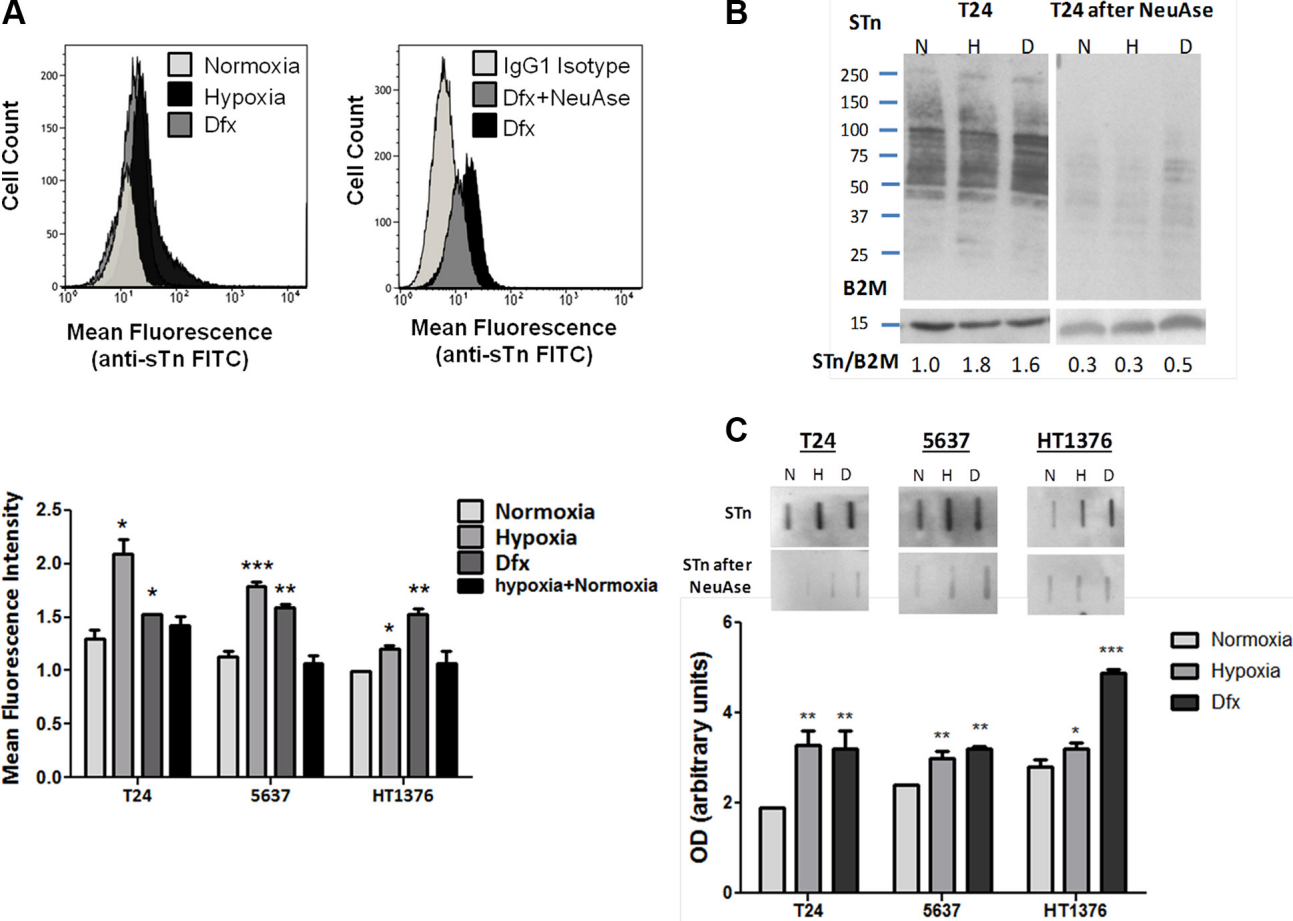
(**A**) STn expression in bladder cancer cell lines (T24, 5637, HT1376) in normoxia (21.0% O_2_), hypoxia (0.1% O_2_; 24 h for T24 and 5637 and 6 h for HT1376), Dfx and Hypoxia followed by reoxygenation (0.1 + 21.0% O_2_). Briefly, approximately 10^4^ cells were probed with the anti-STn TKH2 mouse monoclonal antibody and analysed by flow cytometry. Negative controls included cells treated with α-neuraminidase (NeuAse), responsible by removing the sialic acid from the STn antigen, thereby impairing recognition by the antibody. An IgG1 isotype was also used as negative control. Accordingly, exposure to hypoxia and Dfx induced a significant increase in STn expression (left FACS and bottom Graph). The specificity of TKH2 monoclonal antibody to STn was confirmed by the significant decrease in the mean fluorescence intensity presented by cells treated with neuraminidase (right FACS). STn increase in Dfx-treated cells suggests these events may be directly and/or indirectly regulated by HIF-1α. The reoxygenation of hypoxic cells restored the basal STn levels, demonstrating the key role played by O_2_ levels in STn expression. (**B**) STn-expressing glycoproteins determined by western blot in normoxia, hypoxia and Dfx for T24 cell line. The western blots highlight that the STn antigen is mostly present in proteins with a molecular weight above 50 kDa. The relative levels of STn are presented bellow for each lane and are the average value of three independent experiments. For each cell line, hypoxia and Dfx-exposed cells presented statistically higher levels of STn in comparison to normoxia when compared to the reference protein (*p* < 0.05 for hypoxia and Dfx), thus in agreement with flow cytometry analysis. Similar tendencies were observed for the other cell lines (data now shown). The loss of signals after NeuAse treatment confirms the specificity of antibody recognition. (**C**) Total STn levels by slotblot in T24, 5637 and HT1376 cells in normoxia, hypoxia and Dfx. A marked increased in STn is observed in hypoxia and Dfx for all cell lines. Again, the specificity of the signals was confirmed by NeuAse treatment. *p < 0.05; **p < 0.01; ***p < 0.001 (Student's *T*-test).

Recently we have described that bladder cancer cell lines express only residual amounts of STn antigen, despite its significant presence in advanced stage tumours [21]. In the present study, our flow cytometry results evidenced that hypoxia and Dfx treatments promoted the overexpression of STn in all cell lines (Figure 3A), which was more pronounced in T24 and 5637 cells under hypoxia (60% increase) and less pronounced in HT1376 cells in hypoxia (20% increase). The presence of STn was confirmed by the decrease in cell fluorescence after neuraminidase treatment (Figure 3A). The reoxygenation of hypoxic cells restored the basal levels of STn expression (Figure 3A), denoting the key role played by oxygen in STn overexpression. STn increase in hypoxia and Dfx was further validated by western blot, which also highlighted that this post-translational modification is present in a broad range of glycoproteins in all cell lines (Figure 3B). Since the alterations in STn expression in hypoxia and Dfx were not unequivocally evident in the western blots, slot blots were performed (Figure 3C). This confirmed flow cytometry observations and also the specificity of antibody recognition, as translated by the loss of signal in neuraminidase digested samples (Figure 3C). A more in depth screening for other cancer associated O-GalNAc short-chain glycoforms by western blot further revealed that these cells did not express the Tn (precursor of STn) and the T antigens in any of the studied conditions. In agreement with these observations, the Tn and T antigens are also rarely detected in bladder tumours, irrespectively of their stage [36]. However, we observed an increase of sialyl-T (ST) in hypoxic and Dfx-exposed T24 and 5637 cells, which was not evident for HT1376 (Supplementary Figure S5). Nevertheless, when compared to STn expression, the ST antigen was mostly present in high molecular weight glycoproteins, denoting a more restricted glycoproteome which warrants validation future studies. Elevations in the ST antigen have been reported in bladder tumours but their contribution to bladder cancer progression and dissemination still warrants clarification [37, 38]. These results suggest that sialylation, in particular of the Tn antigen, may be amongst the key events driving the premature stop in O-glycosylation extension in a wide number of membrane glycoproteins.

We have further evaluated the transcription of *ST6GALNAC1*, the gene encoding for ST6GalNAc-I, the glycosyltransferase responsible by the sialylation of the Tn antigen, originating STn. As shown in Supplementary Figure S6A, *ST6GALNAC1* mRNA levels presented an increase in hypoxic and Dfx-exposed cells compared to normoxia. Of note, *ST6GALNAC1* was a low transcription gene (2–4 *ST6GALNAC1* molecules per million of reference gene molecules), thus in accordance with the low levels of STn presented by established cell lines. Even though *ST6GALNAC1* amplification was carried out near the limit of quantification of the technique, we emphasize that increased transcription was consistently observed in hypoxic and Dfx-exposed cells for all cell lines (Supplementary Figure S6). These differences were more notorious and statistically significant when samples from the same condition were taken together, irrespectively of the cell line (Supplementary Figure S6A). Furthermore, the reoxygenation of hypoxic cells and the removal of Dfx from the culture medium restored *ST6GAlNAC1* transcript levels and decreased STn expression to normoxic levels in all cell lines, reinforcing that the variations in *ST6GAlNAC1 mRNA* levels were the result of experimental conditions. Altogether, these observations suggest a possible upregulation of this gene, which warrants future confirmation in cancer cells overexpressing this antigen. Despite its low transcription levels, we could confirm the presence of ST6GalNAc-I by western blot (Supplementary Figure S6B). Accordingly, the western blot highlights two main bands (bellow 75 kDa and near 50 kDa) derived from the full length protein and a shorter protein isoform, which is still a fully functional glycosyltransferase capable of inducing STn expression [39]. Nevertheless, we could not confirm by western blot the increase in ST6GalNAc-I suggested by transcripts analysis. Further studies should be undertaken using models with higher *ST6GALNAC1* levels to fully disclose the role of hypoxia in this context.

We have further investigated the expression of *C1GALT1*, which encodes the glycosyltransferase competing with ST6GalNAc-I for the extension of the Tn antigen, originating the T antigen; of *GCNT1* encoding the C2GnT that further elongates the T antigen originating core 2; and of *ST3GAL1* responsible by ST antigen biosynthesis and consequent stop in O-glycosylation extension (structures and results detailed in Supplementary Figure S6). We have noted a mild increase in *C1GALT1* and a striking downregulation of *GCNT1* in hypoxia and Dfx, particularly in T24 and 5637 cell lines. A mild increase in *ST3GAL1* was also observed for all cell lines under hypoxia and Dfx. Even though our work focuses primarily on the STn antigen, whose biological significance is known in bladder cancer [21, 23], these findings reinforce the notion that hypoxia decisively contributes to stop protein O-glycan extension at the cell surface beyond the T antigen. Furthermore, it highlights the key role of sialylation in this process, in what appears to be an HIF-1α mediated event that warrants future clarification.

### Glycoproteomics of hypoxic cells

**Figure 4:**
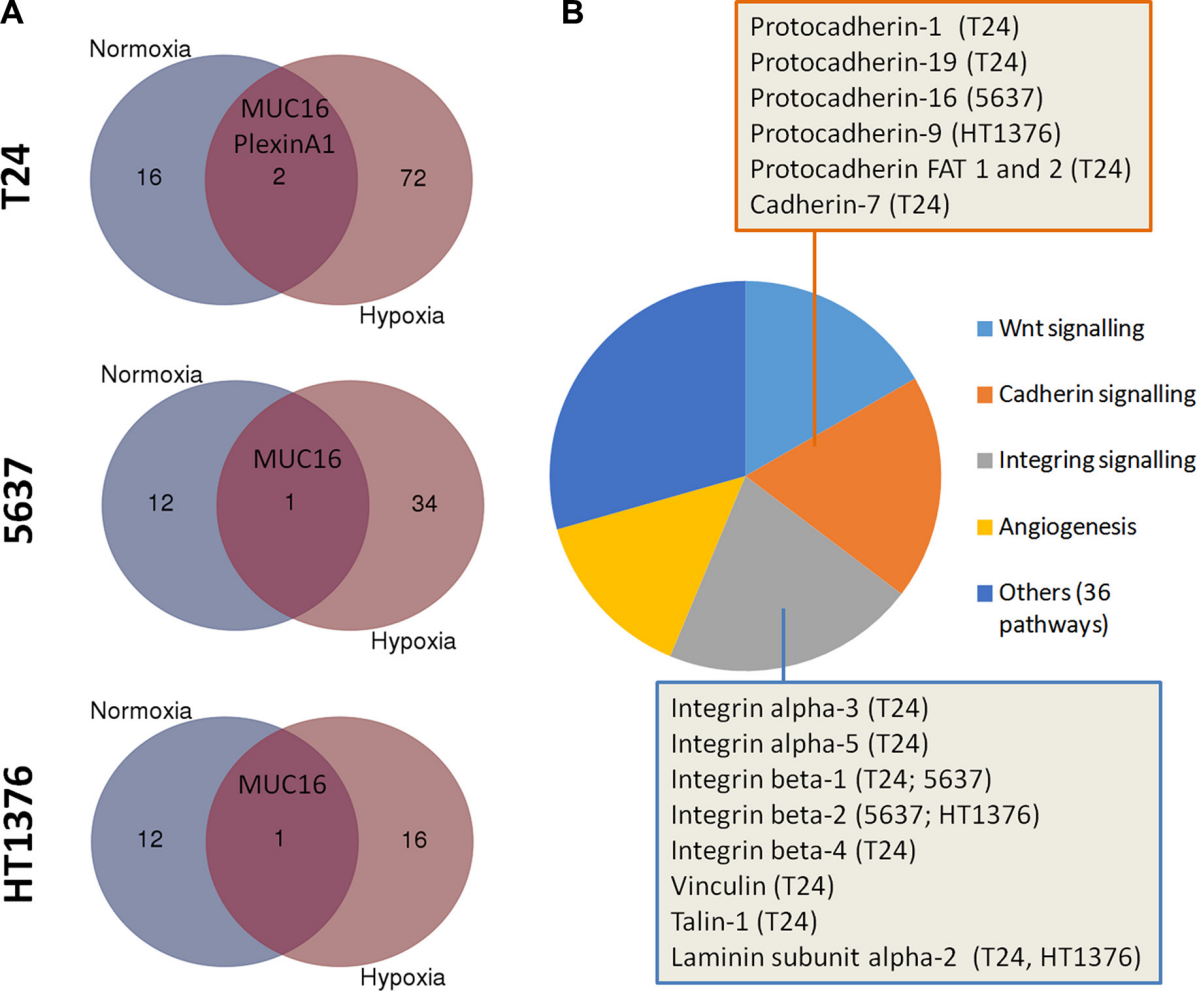
(**A**) Number of putative STn-expressing glycoproteins identified by VVA lectin affinity-nanoLC tandem ESI-MS in normoxic and hypoxic cells. Briefly, proteins isolated from T24, 5637 and HT1376 cell lines in normoxia and hypoxia were digested with α-neuraminidase, which removes the sialic acid from STn exposing the Tn antigen. The samples were then enriched for Tn-expressing glycoproteins by VVA affinity chromatography and identified by nanoLC tandem ESI-MS. Since the Tn antigen was not detected in the original samples (before neuraminidase treatment) by both western blot and flow cytometry, it is likely that the glycoproteins identified by this approach may yield the STn antigen. Accordingly, a higher number of putative STn-expressing glycoproteins were present in hypoxic cells in comparison to normoxia. Interestingly, MUC16 was the only glycoprotein common to all cells and conditions. (**B**) Main signalling pathways associated with STn-expressing glycoproteins in hypoxia. Over 35% of the identified glycoproteins were associated with integrin or cadherin signalling pathways, as highlighted in the Figure. Other key oncogenic pathways also included Wnt signalling and angiogenesis. The STn antigen was also found in glycoproteins involved in other 36 molecular functions, highlighting the broad span of biological roles of STn expressing glycoproteins.

A glycoproteomic screening was performed to bring light on the biological significance of STn overexpression in hypoxia. Briefly, STn expressing glycoproteins were isolated by Vicia villosa (VVA) lectin affinity chromatography for the Tn antigen after neuraminidase treatment, and identified by nanoLC LTQ obritrap tandem mass spectrometry (as summarized by Supplementary Figure S7). Even though the low expression of STn was a major limitation, we were able to identify 18 membrane glycoproteins yielding potential O-glycosylation sites in T24 cells and 13 in 5637 and HT1376. Noteworthy, the only common protein amongst the three cell lines in all conditions was MUC16, whose role in bladder cancer remains still poorly understood (Figure 4A, Supplementary Figures S8 and S9; Supplementary Tables S1–S3). However, increased MUC16 levels in serum (CA125 test) have been associated with worst prognosis in bladder cancer [40], suggesting it as part of a malignant phenotype [41]. In hypoxic conditions, the number of identified glycoproteins was significantly higher in all cell lines (72 in T24;34 in 5637; 16 in HT1376; Figure 4A) in comparison to normoxia, thus in accordance with the results obtained by flow cytometry and western blot (Figure 3A and 3B). The identified glycoproteins included different types of integrins and (proto)cadherins (Figure 4B; Supplementary Tables S1–S3), which are known to play a critical role in cell adhesion. More importantly, a higher number of these classes of glycoproteins were found in T24 and 5637 cells, presenting more motile phenotypes. Particularly, we have identified an increase in STn-integrin beta-1 (STn-CD29; Supplementary Figure S9), an integrin glycoform previously recognized as an inducer of cancer cell invasion [42–44]. This was observed for hypoxic T24 and 5637 but not HT1376 cells (Supplementary Figure S9), and was mostly driven by an increase in low molecular weight integrin glycoforms, which likely present a higher number of short-chain O-glycans, including the STn antigen (described in detail in Supplementary Figure S9). Based on a functional protein association network constructed using STRING bioinformatics tool [45] we have also concluded that CD29 is a key hub protein interacting with several other STn-expressing glycoproteins in both cell lines (Supplementary Figure S10). Furthermore, gene ontology analysis using PHANTER revealed that STn-expressing glycoproteins were mostly involved in integrin, cadherin and Wnt signalling pathways (Figure 4B). Notwithstanding, STn expressing glycoproteins in hypoxic 5637 cell line spanned a broader range of biological functions in comparison to T24 and HT1376, with emphasis on angiogenesis and immune modulation (data not shown). In summary, under hypoxia, the STn antigen was mainly found in glycoproteins involved in cell adhesion, denoting a possible involvement in the modulation of cell motility. Moreover, the STn-glycoproteome varied significantly between cell lines, highlighting significant molecular heterogeneity. Future studies should now focus on understanding the influence of STn expression on different glycoprotein functions. Based on these data, particular emphasis should be given to integrin beta-1.

### Hypoxic regulation of cell invasion/migration: exploring glycosylation for selective inhibition

**Figure 5:**
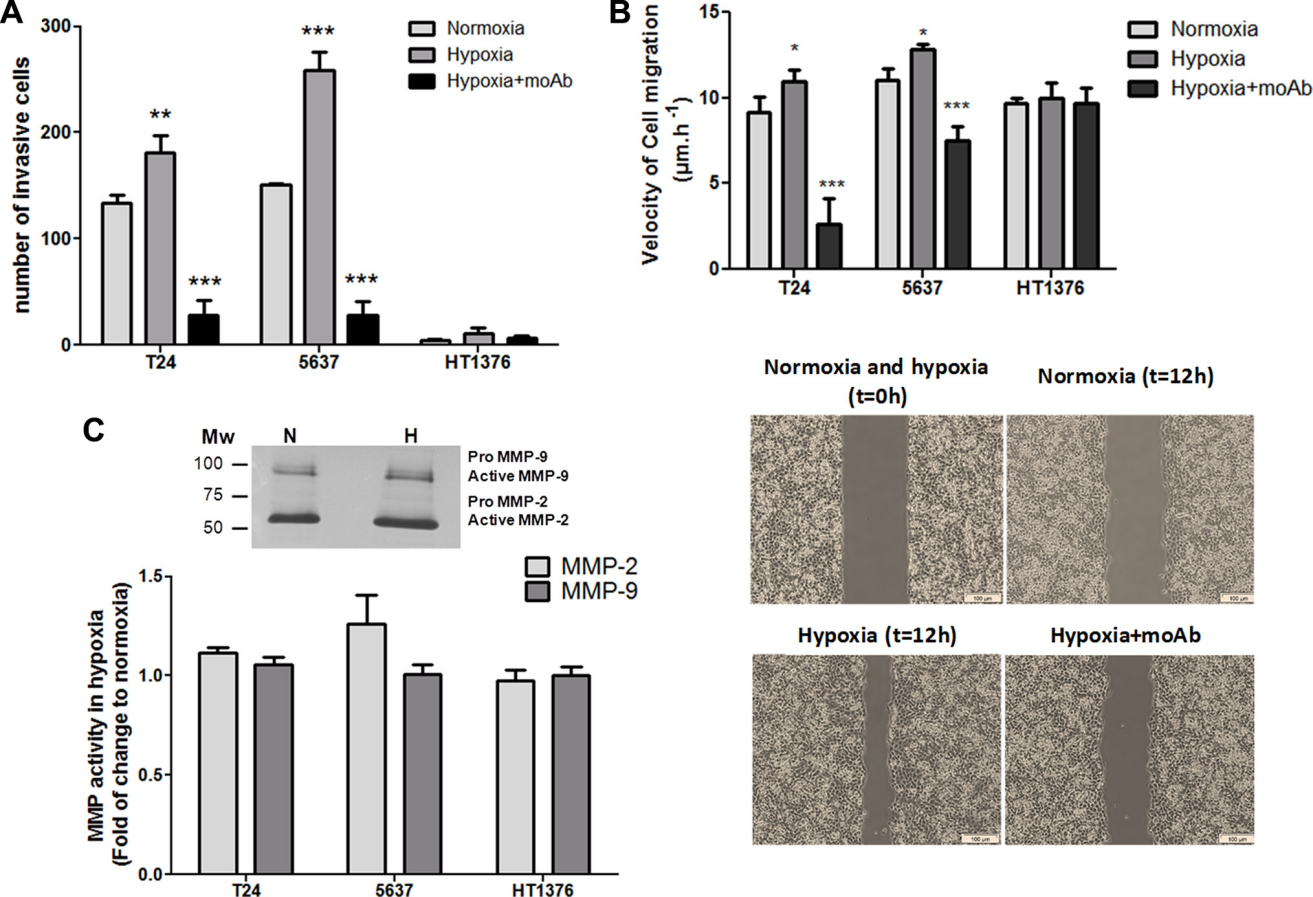
Hypoxia enhances the invasion and migration capability of bladder cancer cells in a STn-dependent manner (**A**) Invasive capability of bladder cancer cells (T24, 5637, HT1376) under normoxia, hypoxia, and hypoxia + anti-STn moAb TKH2. T24 and 5637 display invasive properties under normoxia, whereas HT1376 showed only residual invasive capability. Exposure to hypoxia significantly increased invasion in T24 and 5637 cell lines. Hypoxic HT1376 cells did not present an invasive phenotype. Exposure to anti-STn moAb TKH2 monoclonal antibody promoted a dramatic decrease in invasion in all cell lines. More importantly, the invasive capability of cells exposed to TKH2 was significantly lower when compared to cells grown in normoxia, where STn is also present, even though in lower levels than in hypoxia. Therefore, we envisage that the anti-STn antibody may inhibit/target cancer cells led to express *de novo* the STn antigen in hypoxia, as well as clones that originally presented the glycan. These results suggest that this antibody may be used to selectively inhibit invasion. (**B**) Exposure to hypoxia increased the migratory capacity of the cells in relation to normoxia, in a STn-dependent manner. T24 and 5637 cell lines increased their migratory capacity under hypoxia, which was significantly inhibited by exposure to TKH2, further suggesting that this antibody may be used to selectively inhibit migration. Noteworthy, since invasion assays were done using wells with defined gaps, *t* = 0 is the same for normoxia and hypoxia. (**C**) MMP activity in hypoxic cells in relation to normoxia determined by gelatinase zymography. Representative gelatinase zymogram of bladder cancer cells show two bands at 72 and 92 kDa corresponding to active forms of MMP-2 and MMP-9, respectively (upper Figure). Residual amounts of inactive pro-MMPs could also be observed. Accordingly, no significant alterations in MMP-2 and -9 activity could be observed (lower Graph) suggesting that MMP-2 and -9 activity is not a key event underlying the higher invasive capability developed by bladder cancer cells under hypoxia. Graphs represent average value of three different replicates and ***p < 0.001; **p < 0.01; *p < 0.05.

Hypoxia has been described to potentiate the migration/invasion of cancer cells, which is a critical step for disease progression and dissemination. Based on these observations, the three bladder cancer cell lines were first characterized in relation to their migration/invasive potential on Matrigel. In normoxic conditions only T24 and 5637 cells presented invasive potential, which significantly increased for both cell lines by exposure to hypoxia (Figure 5A) On the other hand, HT1376 cells did not show an invasive phenotype, even when exposed to hypoxia. These observations are consistent with the less cohesive and more motile phenotype suggested by the morphological evaluation of hypoxic and Dfx-treated T24 and 5637 cells, as well as their more pronounced mesenchymal-like characteristics. Notably, the reoxygenation of hypoxic cells decreased cell invasion to the levels found in normoxia (data not shown), highlighting the key role played by oxygen levels. In agreement with these observations, hypoxic T24 and 5637 cells also showed increased migration capability compared to normoxia (Figure 5B).

Gelatine zymography was then used to estimate the contribution of matrix metalloproteases MMP-2 and -9 activities to hypoxia-induced cell invasion, since these proteases have been extensively implicated in bladder cancer progression [46, 47] (Figure 5C). Interestingly, we observed similar MMPs activity for the three cell lines under normoxia, with only a slight increase (not statistically significant) in MMP-2 activity under hypoxia, despite marked differences in invasion. These results suggest that the higher invasion presented by hypoxic T24 and 5637 cells is not related with increased MMP-2 and MMP-9 activity. It is likely that other factors such as morphological changes and alterations in cell adhesion may increase cell motility, as suggested by migration assays (Figure 5B) and enhanced invasion.

Recently we have demonstrated, using glycoengineered cell models, that STn expression enhanced the migratory and invasive potential of bladder cancer cells [20, 21]. Similar observations were made for gastric [24], colon [48] and breast [49, 50] cancer cells, highlighting its key pancarcinomic role played in invasion. We now hypothesize that STn overexpression may be amongst the molecular events driving increased migration and invasion in hypoxic conditions, as suggested by glycoproteomic analysis. Taking into account these observations, we have selectively targeted STn-expressing cells with the anti-STn TKH2 monoclonal antibody (Figure 5A and 5B). Despite the key role of STn in several oncogenic pathways, the presence of the anti-STn monoclonal antibody had no impact on cell viability; however it significantly inhibited cell invasion and migration capacity, suggesting that these cellular processes occurred on a STn-dependent manner.

In summary, our data shows that hypoxia enhanced bladder cancer cell invasion in cells presenting more marked mesenchymal-like properties, in what appears to be a STn-dependent event. Furthermore, and more importantly, it demonstrates that targeting the STn antigen may provide the necessary means to circumvent the invasive potential of bladder cancer cells.

### Expression of hypoxia markers and STn in bladder tumours

The expressions of HIF-1α and cancer-associated glycan STn were evaluated by immunohistochemistry in a series including 30 non-muscle invasive bladder cancer (NMIBC) and 42 muscle invasive bladder cancer (MIBC), representing all stages of the disease (pTa, pT1, pT2, pT3, pT4), using human surgical resections in order to disclose possible associations between altered glycosylation and hypoxia.

**Figure 6:**
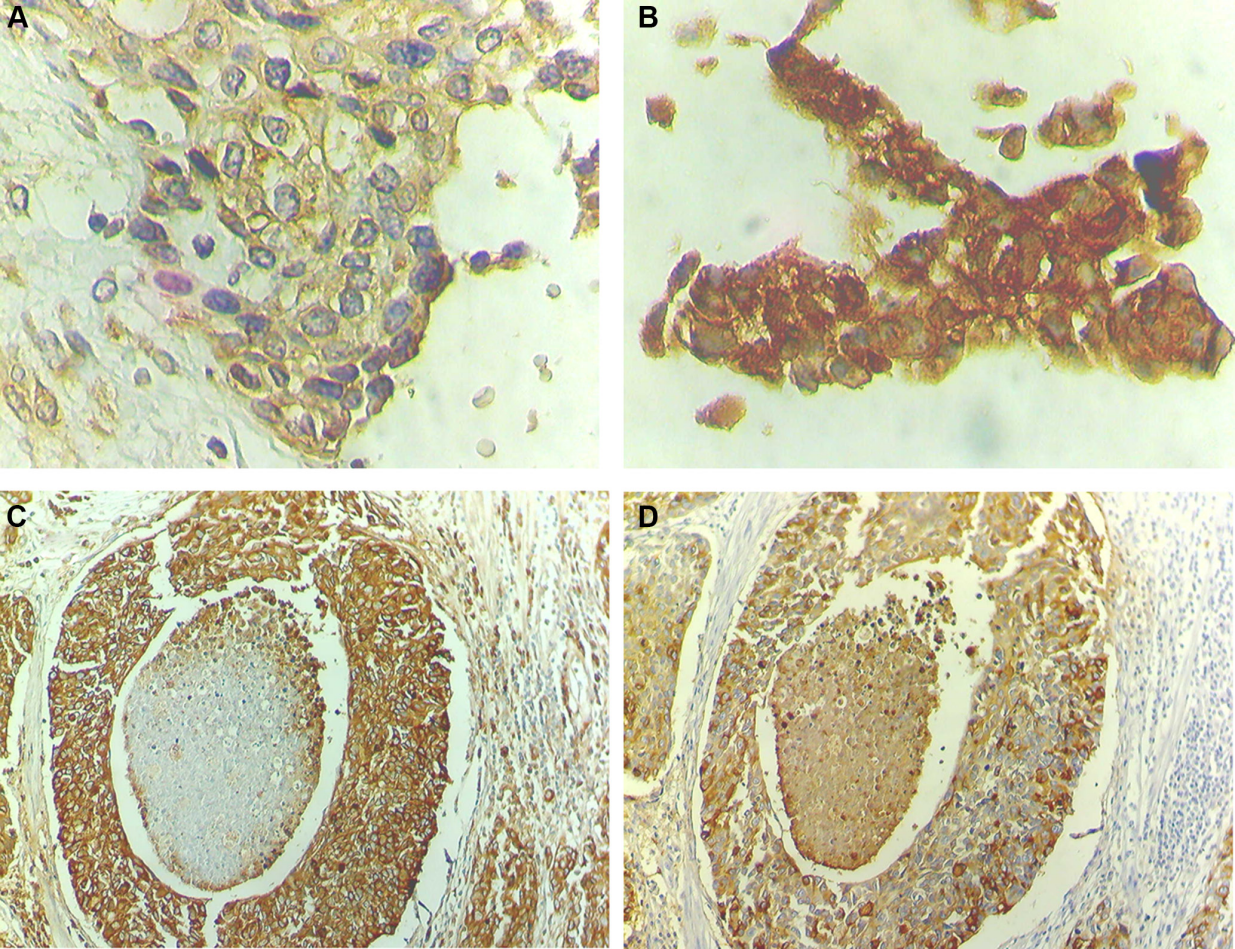
STn overexpression co-localizes with high nuclear HIF-1α expression areas HIF-1α presents mainly a cytoplasmic expression and strong diffused expression troughout the cell when nuclear staining is present. (**A**) Advanced stage bladder tumour showing low levels of nuclear HIF-1α (low hypoxic tumour; 200× magnification). (**B**) Advanced stage bladder tumour showing high levels of nuclear HIF-1α (high hypoxic tumour; 200× magnification). (**C**) Advanced stage bladder tumour area showing high STn expression (100× magnification). (**D**) STn-positive advanced stage bladder tumour area with high nuclear HIF-1α expression (100× magnification).

Notably, all studied tumours were positive for HIF-1α, with approximately 70% presenting an extensive expression (> 20% of the tumour area), irrespectively of their stage. HIF-1α was predominantly detected in the cytoplasm but also in the nucleus, where it acts as a transcription factor. In particular, 43% of NMIBC (13/30) and 85% of MIBC (37/43) presented both cytoplasmic and nuclear HIF-1α expressions, which demonstrates an association between nuclear expression and advanced stage bladder cancer (*p* < 0.05). These tumours were further re-classified as low and highly hypoxic based on the percentage of nuclear HIF-1α expression (Figure 6A–6B; Table 1). Accordingly, high nuclear HIF-1α expression was observed in 43% of the NMIBC (pTa and pT1) and 72% of the MIBC (pT2, pT3 and pT4), reinforcing the association between this phenotype and tumour muscle invasion (*p* < 0.05).

**Table 1:** Nuclear HIF-1α phenotype in bladder tumours in different stages of the disease

Tumour Stage	Tumour hypoxia based on nuclear HIF-1α				*χ*^2^
	Low	High	Low	High	
**Ta (15)**	11 (73%)	4 (27%)	NMIBC	NMIBC	*p* < 0.05
**T1 (15)**	6 (40%)	9 (60%)	17 (57%)	13 (43%)	
**T2 (13)**	4 (31%)	9 (69%)			
**T3 (15)**	5 (33%)	10 (67%)	MIBC	MIB C	
**T4 (15)**	3 (20%)	12 (80%)	12 (28%)	31 (72%)	
**Total (73)**	29 (40%)	44 (60%)			

The STn antigen was mainly present at the cell surface and, in less extent, in the cytoplasm. It presented a focal expression throughout the tissues that did not exceed 30% of the tumour area, being frequently observed at tumour invasion fronts. According to Table 2, the STn antigen was also mainly expressed by MIBC (60%) when compared to NMIBC (30%), which associates the antigen expression with muscle invasion (*p* = 0.03), reinforcing the results previously described by us [21, 36]. This association further reinforces that STn may play a role in tumour invasion, as suggested by studies *in vitro*. We have also observed that the STn antigen co-localized with tumour areas of high nuclear HIF-1α expression (Figure 6C–6D), even though it could also be found in non-hypoxic areas, suggesting that other factors may also lead to STn overexpression in tumours.

**Table 2:** STn expression in bladder tumours according to stage and co-localization with hypoxic tumour areas

Tumour Stage	STn Expression		*χ*^2^	Co-localization with nuclear HIF-1^a^
**Ta (15)**	3 (20%)	NMIBC	***p* < 0.05**	3/4 (75%)
**T1 (15)**	6 (40%)	14 (33%)		4/6 (67%)
**T2 (13)**	7 (50%)	MIBC		3/4 (75%)
**T3 (15)**	10 (67%)	26 (60%)		7/9 (77%)
**T4 (15)**	9 (60%)			2/3 (67%)
**Total (73)**	34 (46%)			19/26 (73%)

## CONCLUDING REMARKS

STn is considered a cancer-specific pancarcinoma antigen, based on the fact that it is expressed by the majority of advanced stage solid tumours, including bladder cancer, while generally absent from the corresponding healthy tissues [15–19, 21]. Nevertheless, the events responsible by the premature stop in *O*-glycans elongation through Tn antigen sialylation in tumours remain to be clarified. To this date, two main mechanisms leading to STn biosynthesis have been described: i) the overexpression of *ST6GALNAC1* [22, 50], which is commonly observed in human carcinomas [15, 50]; ii) loss-of-function of *C1GALT1* chaperone Cosmc [51, 52], which is considered a rare event [53]. However, epigenetic changes such as hypermethylation of *Cosmc* [54], alterations in pH [55], and relocation of GalNAc-transferases from the Golgi to the endoplasmic reticulum [56] may also account for a premature stop in O-glycosylation. In the present work, we have demonstrated, for the first time, that hypoxia, a salient feature of advanced stage bladder tumours [13, 14], can also induce glycosyltransferase expression alterations that favour the expression of simple mucin-type sialylated *O*-glycans. We have observed that, under oxygen deprivation, bladder cancer cells overexpressed classical hypoxia markers, such as HIF-1α and CA-IX, switched to anaerobic metabolism and presented significant morphological alterations towards a less cohesive phenotype, thus in accordance with findings from other models [5, 6]. Concomitantly, all cell lines overexpressed the STn antigen, irrespectively of their genetic and molecular background, highlighting a ubiquitous mechanism for bladder cancer cells under hypoxia. These observations reinforce preliminary findings in patient-derived xenografts supporting the key role played by the tumour microenvironment in STn overexpression [23]. Microenvironmental factors may also account for the remarkably low levels of STn found in several cell models derived from tumours which are known to overexpress the antigens. However, our flow cytometry and gene expression data suggests that only rare clones are endowed with the capability to overexpress the STn antigen when exposed to hypoxia. Efforts are ongoing in order to isolate these cells for molecular and functional characterization, envisaging more insights about the role of STn-expressing cells in cancer.

We have also observed that hypoxia significantly alters the expression of key enzymes involved in the initial steps of protein O-glycosylation, with emphasis on the downregulation of *GCNT1*, as well as the overexpression of *ST3GAL1* and possibly *ST6GALNAC1*, which would explain the accumulation of short-chain sialylated O-glycans at the cell surface. Since a similar behaviour was observed in Dfx-treated cells, we hypothesized that the extension of *O*-glycans may directly or indirectly be regulated by HIF-1α. However, since Dfx interferes with several cell processes [57, 58], future validations should include Knock down/out models. Interestingly, we have previously described that STn overexpression was associated with the overexpression of *ST6GalNAc-I* in advanced stage bladder tumours [20, 21], which are frequently highly hypoxic [59, 60]. However, even though RT-PCR analyses of all bladder cancer cells suggested an increase in *ST6GALNAC1* transcription in hypoxia and Dfx, these observations could not be validated by western blot. This most likely stems from the low sensitivity of the technique for mild alterations occurring in low expressed proteins. Given the difficulty in mimicking the tumour environment responsible by STn overexpression *in vitro*, wich often translates in low STn levels in cell lines comparison to human tumours, this feature should be readdressed in relevant STn-overexpressing bladder cancer models, namely patient-derived xenogfrats. Genetic and epigenetic changes regulating glycosyltransferases and its chaperones, alterations in sugars biosynthesis pathways, glycosyltransferases relocation within the secretory pathways and proteome modulation, among other factors may also contribute to O-glycome alterations under hypoxia and should also be carefully accessed in future studies. As such, focus should now be set on defining the events underlying STn expression in hypoxia in order to design novel therapeutics.

Another important finding is that oxygen levels act as an on-off switch for cell migration/invasion, in a mechanism that appears to be independent of MMP-2 and -9 activities. Based on our observations and previous reports, we hypothesize that antagonization of O-glycosylation extension, including STn overexpression, may be amongst a wide array of molecular events driving more mesenchymal bladder cancer cells towards invasion. Interestingly, STn increase in cells with more epithelial nature (HT1376) did not translate in increased invasion, denoting a dependence on the cells specific proteome and other molecular features. Probably the reduction in O-glycans polymerization may translate into significant conformational changes in cell-surface proteins, thereby affecting adhesive properties. In agreement with this hypothesis, we have found the STn antigen in key glycoproteins involved in cell-cell and cell-extracellular matrix adhesion, namely MUC16, integrins, cadherins, thus in agreement with previous reports [20, 24, 61–64]. This is likely to induce major alterations in integrin and cadherin-mediated signalling, as suggested by bioinformatics analysis, translating into major alterations in morphology and cell behaviour, as previously reported for other models [7]. Furthermore, the overexpression of the STn antigen by glycoengineered bladder [21], gastric [24] and breast cancer [49, 65] cell models has consensually demonstrated to result in enhanced migration/invasion. Previous studies have also pointed out STn as a biomarker of epithelial-to-mesenchymal transition, a crucial milestone not only to invasion but also to metastasis. Namely, Lin et al. observed high levels of STn in mesenchymal cells isolated from a bone marrow biopsy of a nasopharyngeal cancer patient [66]. Furthermore, the STn antigen has been detected both in primary tumours and in the metastasis of different human carcinomas [16, 17, 19]. Supporting these observations and the association of STn with hypoxia, we have also found the STn antigen in highly hypoxic bladder tumour areas, including invasion fronts, which are enriched in mesenchymal cells [67, 68]. Moreover, the analysis of patient samples also confirmed our previous reports [21] linking STn overexpression to invasive bladder tumours and decreased overall survival [21, 36].

**Figure 7:**
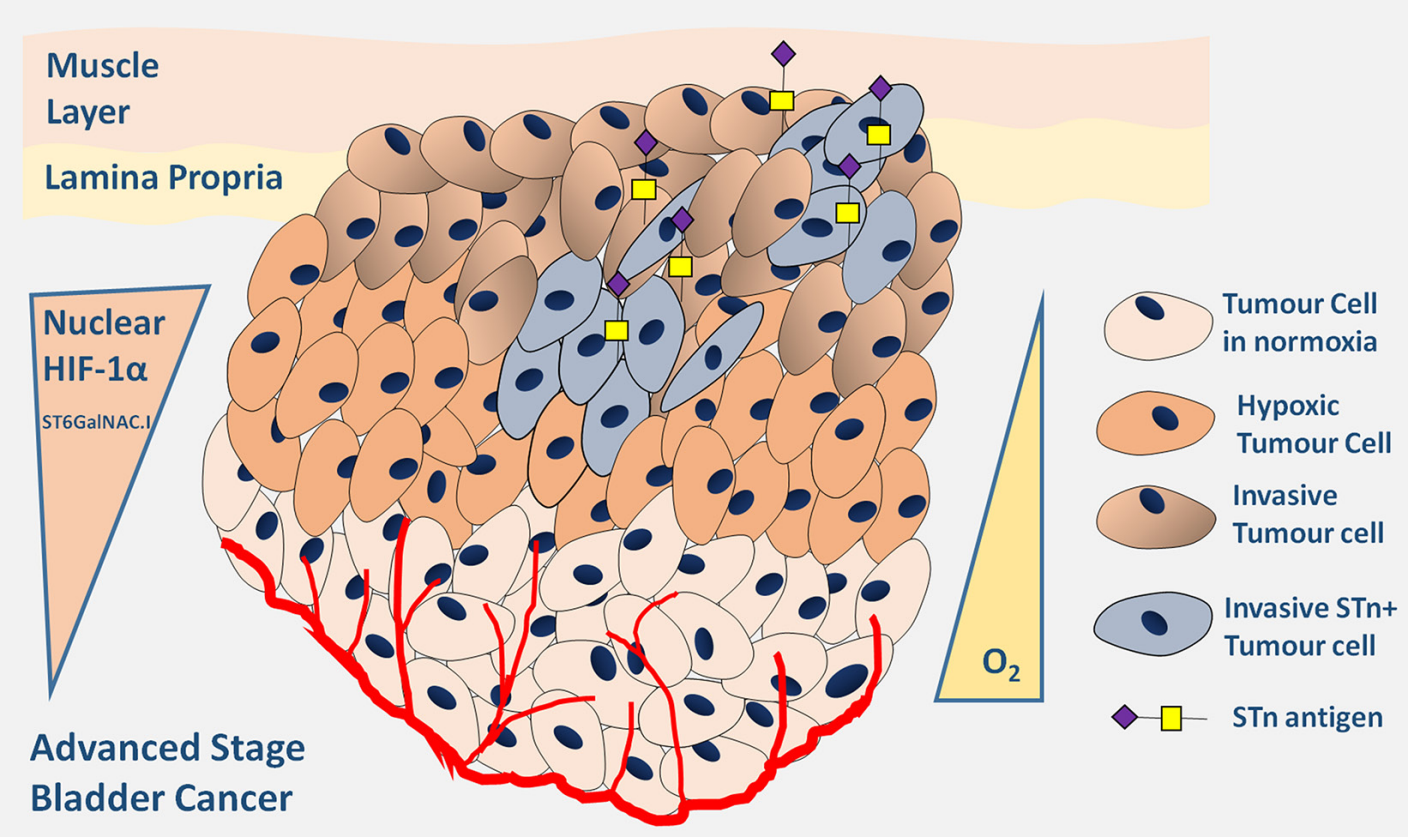
Schematic representation of advanced stage hypoxic tumours showing increased STn expression Accordingly, the Figure highlights the existence of a subpopulation of hypoxic cells showing high nuclear HIF-1α expression and also STn expression. These cells are endowed with the capability of invading the basal and muscle layers contributing to poor outcome.

In conclusion, we have demonstrated that antagonization of O-glycosylation extension and, in particular STn overexpression, are surrogate markers of malignant alterations in the bladder associated with hypoxia. Furthermore, we have identified a new glycosylation-mediated mechanism used by bladder cancer cells to adapt to the microenvironmental challenge posed by hypoxia, underlined by Figure 7. Hypoxic bladder cancer cells, possibly endowed with active stem cell and/or mesenchymal programs, repress the *O*-glycosylation of cell-surface proteins by downregulating key enzymes involved in the extension of the glycans. These events translate into the overexpression of the STn antigen that potentially contributes to potentiate cell invasion. More importantly, we have demonstrated that targeting the STn antigen with a monoclonal antibody may provide the necessary means to circumvent invasion, thereby establishing the molecular basis for future therapeutic development. A more in depth comprehensive characterization of bladder tumours-specific STn subproteome may now allow the design of highly specific therapeutic strategies against hypoxic niches, which harbour more malignant cells. As such, this study has contributed to create the rationale for glycan-based therapeutics, currently under development in our laboratory, against advanced stage bladder tumours, which remain “orphan diseases” regarding the introduction of novel therapeutics [69].

## MATERIALS AND METHODS

### Patients and sampling

Representative formalin-fixed paraffin-embedded surgical specimens were prospectively obtained from 73 patients with urothelial bladder carcinomas whom underwent transurethral resection of non-muscle invasive bladder tumours (NMIBC; 15 pTa and 15 pT1) or radical cystectomy for muscle invasive bladder cancer (MIBC; 13 pT2, 15 pT3; 15 pT4), between July 2011 and May 2012. The median age of the patients was 70 years (range 41–86); fifty-three (72.6%) were male and twenty (27.4%) were female. None of these patients had received prior adjuvant therapy.

All procedures were performed with the approval of the Ethics Committee of IPO-Porto, after patients' informed consent and strictly following the principles of the Helsinki declaration. All clinicopathological information was obtained from patients' clinical records.

### Cell lines and culture conditions

The T24 (grade III), 5637 (grade II) and HT1376 (grade III) bladder cancer cell lines used in this work were acquired from DSMZ (Düsseldorf, Germany) and recently characterized and authenticated by our group [33]. Accordingly, the T24 cell line is representative of the FGFR3/CCND1 disease progression pathway, presenting a mutated HRAS and over-expression of CCND1. The 5637 and HT1376 cells represent the E2F3/RB1 pathway with loss of one RB1 copy and mutation of the remaining copy. Additionally, HT1376 cells exhibit PTEN gene deletion and no alterations in PIK3CA, which in combination with the inactivation of p53, translates into a more invasive and metastatic potential. In contrast, the 5637 cell line presents PIK3CA gene deletion and no PTEN alterations, which translates into a less-invasive phenotype.

The cells were cultured in RPMI 1640+GlutaMAXTM-I medium (Gibco, Life Technologies), supplemented with 10% heat-inactivated FBS (Gibco, Life Technologies) and 1% penicillin-streptomycin (10,000 Units/mL penicillin; 10,000 mg/mL streptomycin; Gibco, Life Technologies). Cell lines were cultured as a monolayer at 37°C in a 5% CO_2_ humidified atmosphere (normoxia), and were routinely subcultured after trypsinization. The cells were also grown under hypoxia at 37°C with a 5% CO_2_, 99.9% N_2_ and 0.1% O_2_ atmosphere in a BINDER C-150 incubator (BINDER GmbH). Additionally, cells were also grown under normoxia in the presence of 500 μM Deferoxamine Mesilate salt CRS (Dfx, Sigma-Aldrich), a HIF-1α pathway stabilizer, used as positive control [25].

### Expression of HIF-1α, CA-IX and ST6GalNA-I

The expression of hypoxic biomarkers HIF-1α and CA-IX, a HIF-1α transcriptionally activated protein [70] and ST6GalNAc-I by the selected cell lines was evaluated by western blot as previously described [20]. The antibodies used were rabbit anti-human HIF-1α clone [16Η4L13] (1:250 in TBS; Invitrogen), mouse anti-human CA-IX clone [2D3] (1:2000 in TBS; Abcam) and mouse anti-human ST6GalNAc-I clone 1C9 [15]. The MCRSTn^+^ cell line glycoengeneired to overexpress the ST6GalNAc-I [21] was used as a positive control for ST6GalNAc-I identification by western blot. B2M expression was used as loading control using anti-beta 2 Microglobulin antibody [EP2978Y] (1:10,000 in TBS, Abcam).

### L-Lactate quantification

The Abcam's fluorometric L-Lactate assay kit (Abcam) was used to determine the concentration of L-Lactate in culture media. Briefly, Lactate was oxidized by lactate dehydrogenase to generate a product which interacts with a probe producing fluorescence. The reaction product fluorescence was measured at Ex/Em = 535/590 nm in a microplate reader (SynergyTM Mx, BioTek).

### Evaluation of cell viability

Cell viability was determined using the Trypan Blue Exclusion Test of Cell Viability. Briefly, cells uptaking trypan blue were considered non-viable. Cell viability was calculated as the number of viable cells divided by the total number of cells within the grids on a Newbauer chamber.

### Evaluation of cell proliferation

Cell proliferation was evaluated with a colorimetric BrdU cell proliferation ELISA kit (ab126556 Abcam) according to the manufacturer instructions. Accordingly, the test estimates the incorporation of Bromodeoxyuridine (5-bromo-2′-deoxyuridine, BrdU), a synthetic nucleoside analogue to thymidine, into newly synthesized DNA of actively proliferating cells. Briefly, the cells (10^4^ cells/mL) were cultured in 96 well plates and BrdU was added to the wells during the final 24 hours of culture. The amount of incorporated BrdU was estimated using an anti-BrdU monoclonal antibody, an horseradish peroxidase-conjugated goat anti-mouse antibody and tetra-methylbenzidine (TMB) as chromogen, all provided by the kit. To enable antibody binding to incorporated BrdU, cells were fixed, permeabilized and the DNA denatured. The reaction was monitored at 450 nm on a microplate reader (SynergyTM Mx, BioTek). Three independent assays were performed and cells were seeded in duplicate for each cell line. Results are presented as mean ± SD for each condition.

### Expression of short-chain O-glycans

The STn expression in cell lines was determined by flow cytometry using the anti-STn mouse monoclonal antibody clone TKH2 [71] as described in Ferreira *et al.* [21, 37]. The expression of the Tn and T antigens were also accessed using 1E3 and 3C9 mouse monoclonal antibodies, respectively [22, 72]. Experiments were conducted using approximately 10^6^ adherent cells, which were converted into single cell suspensions using Versene (Gibco) at 4°C, followed by filtration using a 70 μm Nylon cell strainer (BD Falcon). A minimum of 10^5^ cells were analysed by flow cytometry. The cell population in this study was gated based in FSC and SSC features to avoid background and debris (corresponding to > 95% of the measured cells in each sample). Whole cells digested with α-neuraminidase were used as negative controls. Briefly, prior to analysis, the single cells were treated with 10 U/mL α-neuraminidase from *Clostridium perfringens* (Sigma) for 90 min at 37°C. Treatment with neuraminidase removes the sialic acid from STn originating the Tn antigen (GalNAc-Ser/Thr), which is not recognized by the TKH2 monoclonal antibody [37, 73]. Experiments using a mouse IgG1 [ICIGG1] Isotype Control (Abcam) were also included as negative controls. Polyclonal rabbit anti-mouse immunoglobulins/FITC (DAKO; F0313) was used as secondary antibody. Two independent experiments were performed in triplicate for each cell line and condition. The expression of Tn, STn and T antigens was also confirmed by western blot and slot blot [23]. Neuraminidase treated samples were used as negative controls. The ST antigen was evaluated by western blot using the 3C9 mouse monoclonal antibody after neuraminidase treatment.

### Gene expression

Gene expression was assessed by quantitative polymerase chain reaction (qPCR) under normoxia, hypoxia and Dfx exposure. This included a group of genes associated with stem cells (*NANOG*, *LIN28A*, *POU5F1*, *KLF9*, *KLF4*, *SOX2*); epithelial (*CDH1*, *DSP*, *EpCAM*); Epithelial-to-mesenchymal transition (*SNAI1*, *SNAI2*, *TWIST1*, *TWIST2*, *ZEB1*, *ZEB2*, *RUNX1*, *RUNX2*, *FN1*); mesenchymal (*CDH2*, *VIM*, *SPARC*) phenotypes as well as glycosyltransferases involved in the initial steps of O-glycosylation *(ST6GALNAC1, C1GALT1, GCNT1, ST3GAL1*) described in Supplementary Table S4. Briefly, total RNA from cultured cells was isolated using TriPure isolation Reagent (Roche). RNA conversion and gene expression analysis was performed as previously described [74]. All reactions were run duplicates. All experiments were performed in triplicate. The mRNA levels were normalized to the expressions of *B2M* and *HPRT*, which were found to be the most stable genes under all studied conditions and for the three cell lines out of a panel of seven reference genes (*HPRT*; *B2M*; *ACTB*-Hs99999903_m1; *18S*-Hs99999901_s1; *GAPDH*-Hs03929097_s1; *TBP*-Hs00427620_m1; *SDHA*-Hs00188166_m1). The relative mRNA levels were calculated using The formula 2^−ΔΔCt^ described by Livak et al. [75]. The efficiency of each primer/probe was above 95% as determined by the manufacturer. Interpretation of biological functions translated by alterations in gene expression under hypoxia in relation to normoxia was done using ClueGO version 2.2.5 [76] and CluePedia plugins version 1.2.5 [77] for cytoscape version 3.3.0 [78].

### Glycoprotein enrichment for glycoproteomics

The proteins were extracted from whole cells (10^7^) with 0.05% RapiGest SF (Waters) in 50 mM ammonium bicarbonate using a sonic probe. The lysates were then digested with α-neuraminidase [10 U *Clostridium perfringens* neuraminidase Type VI (Sigma)] to release the sialic acid and expose the Tn antigen. Protein lysates were then loaded on 300 μl Vicia villosa agglutinin (VVA) agarose (Vector laboratories) columns to enrich the extracts in Tn-expressing glycoproteins. The columns were then washed with 10 column volumes (CV) of 0.4 M Glucose in LAC A buffer (20 mM Tris-HCl pH 7.4, 150 mM NaCl, 1 M Urea, 1 mM CaCl_2_, MgCl_2_, MnCl_2_, and ZnCl_2_) followed by 1 ml 50 mM NH_4_HCO_3_. The glycoproteins were then eluted by 4× 500 μl 0.05% RapiGest SF (Waters) with heating to 90°C for 10 min. The glycoprotein fraction was then directly reduced with 5 mM DTT (Sigma) for 40 min at 60°C, alkylated with 10 mM iodoacetamide (Sigma) in dark for 45 min, and digested with trypsin (Promega).

### Nano LC-ESI-MS/MS

A nanoLC system (Dionex, 3000 Ultimate nano-LC) was coupled on-line to a LTQ-Orbitrap XL mass spectrometer (Thermo Scientific) equipped with a nano-electrospray ion source (Thermo Scientific, EASY-Spray source). Eluent A was aqueous formic acid (0.2%) and eluent B was formic acid (0.2%) in acetonitrile. Samples (20 μl) were injected directly into a trapping column (C18 PepMap 100, 5 μm particle size) and washed over with an isocratic flux of 95% eluent A and 5% eluent B at a flow rate of 30 μl/min. After 3 minutes, the flux was redirected to the analytical column (EASY-Spray C18 PepMap, 100 Å, 150 mm × 75μm ID and 3 μm particle size) at a flow rate of 0.3 μl/min. Column temperature was set at 35°C. Peptide separation occurred using a linear gradient of 5–40% eluent B over 117 min., 50–90% eluent B over 5 min. and 5 min. with 90% eluent B. In order to favour the separation and identification of peptides presenting high hydrophobicity, samples were also analysed with a two-step gradient protocol: 5–35% eluent B over 37 min., 35–65% eluent B over 80 min., followed by 65–90% eluent B over 5 min. and 5 min. with 90% buffer B. The mass spectrometer was operated in the positive ion mode, with a spray voltage of 1.9 kV and a transfer capillary temperature of 250°C. Tube lens voltage was set to 120 V. MS survey scans were acquired at an Orbitrap resolution of 60,000 for an m/z range from 300 to 2000. Tandem MS (MS/MS) data were acquired in the linear ion trap using a data dependent method with dynamic exclusion: The top 6 most intense ions were selected for collision induced dissociation (CID). CID settings were 35% normalized collision energy, 2 Da isolation window 30 ms. activation time and an activation Q of 0.250. A window of 90 s was used for dynamic exclusion. Automatic Gain Control (AGC) was enabled and target values were 1.00e+6 for the Orbitrap and 1.00e+4 for LTQ MSn analysis. Data were recorded with Xcalibur software version 2.1.

### Glycoprotein identification and data mining

Data were analysed automatically using the SequestHT search engine with the Percolator algorithm for validation of protein identifications (Proteome Discoverer 1.4, Thermo Scientific). Data were searched against the human proteome obtained from the SwissProt database on 22/11/2015, selecting trypsin as the enzyme and allowing for up to 2 missed cleavage sites and a precursor ion mass tolerance of 10 ppm and 0.6 Da for product ions. Carbamidomethylcysteine was selected as a fixed modification while oxidation of methionine (+15.99491), and modification of serine and threonine with HexNac (+203.07937 u) was defined as variable modification. For whole tumor proteome analysis, only high confidence peptides were considered. In glycoproteomics studies, due to the high lability of the sugar moieties under CID conditions, and the consequent difficulty in identifying modified peptides, Sequest results of low confidence peptides were also considered. Protein grouping filters were thus set to consider PSMs with low confidence and ΔCn better than 0.05. The strict maximum parsimony principle was applied. A protein filter counting peptides only on top scored proteins was also set. Peptides were filtered for Xcorr ≥ 1.5 and ΔCn ≤ 0.05. Cell membrane proteins with at least 1 annotated glycosylation site were selected and the modifications were validated manually. Membrane proteins were sorted in relation to O-glycosylation sites using NetOGlyc version 4.0 [79] to generate the final protein list. Protein molecular and biological functions were interpreted using PHANTER (Protein ANalysis THrough Evolutionary Relationships) [80] and Protein-Protein interactions explored using STRING v10 [45].

### Validation of STn-Integrin beta 1 glycoforms by western blot

Neuraminidase digested glycoproteins from normoxia and hypoxia exposed cells were loaded on 300 μl Vicia villosa agglutinin (VVA) agarose (Vector laboratories) columns and eluted as previously described by us [62]. The isolated glycoproteins were then screened for integrin beta-1 by western blot using rabbit monoclonal anti-integrin beta-1 (Abcam, EP1041Y). Non-neuraminidase treated glycoproteins were used as controls.

### Invasion assay

Invasion assays were performed using BD Biocoat MatrigelTM invasion chambers as described in Ferreira *et al.* [21]. Three independent assays were performed and cells were seeded in duplicate for each cell line. Results are presented as mean ± SD for each condition. Experiments to disclose the role of the STn antigen in invasion were conducted in the presence of anti-STn mouse monoclonal antibody TKH2. Invasion assays with cells incubated with the same IgG1 isotype control used for flow cytometry were used as controls. Triplicate experiments were performed for each cell line and condition.

### Migration assay

Wound healing assays were performed using specific wound assay chambers (Ibidi) containing silicone inserts delimiting a fixed gap of 500 μm. The cells were first seeded into the assay chambers and after 24 h the silicone inserts were removed to allow the cells to migrate. The cells were then incubated with fresh culture medium under normoxia, hypoxia or hypoxia in the presence of anti-STn monoclonal antibody TKH2 for 16 h, corresponding to full gap closure. The cells were monitored every 2 h under a high-resolution inverted microscope (Olympus CKX41). Images were captured with an Olympus camera (Olympus SC30). For assay analysis, cells were tracked using the manual tracking software component of the ImageJ programme. After tracking, the cell paths were analysed using the ‘Chemotaxis and Migration Tool’, a free ImageJ plugin provided by Ibidi to compute center of mass (center of mass of all endpoints) and accumulated distance (mean distance of all cell paths). The results were expressed in terms of mean migration velocity (μm.h^−1^) resulting from three independent replicates.

### Gelatine zymography

Gelatine zymography was performed to determine matrix metalloproteinases (MMP) activity in cells under normoxia, submitted to hypoxia and exposed to Dfx. Proteins (15 μg/lane) from conditioned media were separated on 10% polyacrylamide zymogram gels with 0.1% gelatine (MERCK) as substrate. After electrophoresis, gels were incubated in 2% Triton X-100 (Sigma) in Milli-Q water for protein renaturation, with gentle agitation for 30 min at room temperature. Subsequently, gels were incubated in MMP substrate buffer (50 mM Tris-HCl, pH 7.5; 10 mM CaCl_2_) overnight with gentle shaking at 37°C. The gels were finally stained with filtered Coomassie blue solution (Sigma) for 30 min and then washed with deionized water until the adequate resolution was obtained. Gelatinolytic bands were observed as white areas against the blue background, and the intensity of the bands was evaluated using the Quantity One Software (Biorad).

### Tissue expressions of HIF-1α and STn

Formalin fixed paraffin embedded (FFPE) tissue sections were screened for HIF-1α and STn by immunohistochemistry using the streptavidin/biotin peroxidase method as described previously [21]. The expressions of HIF-1α and STn were evaluated using the above mentioned monoclonal antibodies.

The tumours were classified as low or highly hypoxic lesions based on the nuclear expression of HIF-1α, according to the criteria defined by Birner et al. [81]. Briefly, a semi-quantitative approach was established to score immunoreactivity based on the intensity and extension of the staining. The extension of nuclear staining was rated as follows: no expression-0 points; < 10% nuclei-1 point; 11–50% nuclei-2 points; 50–80% nuclei-3 points, > 81% nuclei-4 points. Staining intensity was rated as follows: weak-1 points: moderate-2 points; strong-3 points. The tumours were then classified in four groups based on the arithmetic sum of these variables: absent (0 points); residual (1–2 points); weak (3); moderate (4–5); and strong (6–7) HIF-1α nuclear expression. For statistical reasons, a two-scale classification was introduced, which allowed classifying the tumours as low hypoxic lesions (weak nuclear HIF-1α immunostaining; 0–3 points) and high hypoxic lesions (moderate to strong nuclear HIF-1α immunostaining; 4–7 points).

The tumours were classified as STn positive, whenever the expression of the antigen was higher than 5%, taking into consideration that the antigen is not expressed by the healthy urothelium [21]. The immunoreactivity was assessed double-blindly by two independent observers (BP and LL) and validated by an experienced pathologist (TA). Whenever there was a disagreement, the slides were revised, and consensus was reached.

### Statistical analysis and data mining

Statistical analysis was performed using the Student's *T*-test for unpaired samples. Differences were considered to be significant when *p* < 0.05. A chi-square test was used to analyse associations between variables and clinicopathological features.

## SUPPLEMENTARY MATERIALS FIGURES AND TABLES






