# Supplementary material for: Hypoxia enhances the malignant nature of bladder cancer cells and concomitantly antagonizes protein O-glycosylation extension
Source: Oncotarget. 2016 Aug 12;7(39):63138–57. doi: 10.18632/oncotarget.11257 (PMC5325352; doi:10.18632/oncotarget.11257)
Supplement: Supplementary file 1 [file oncotarget-07-63138-s001.pdf]

Hypoxia enhances the malignant nature of bladder cancer cells and concomitantly antagonizes protein *O*-glycosylation extension

Supplementary Materials

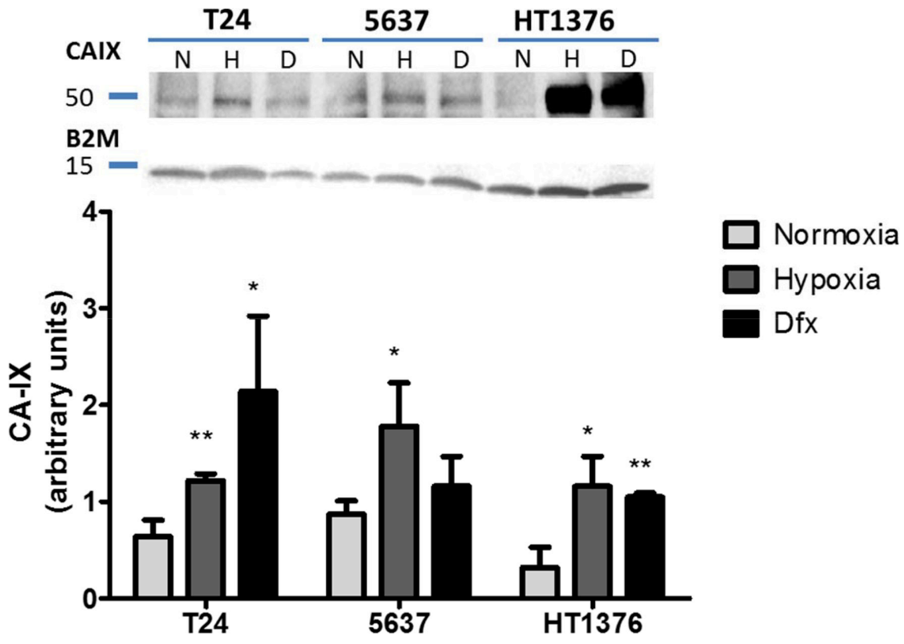

Supplementary Figure S1: Levels of HIF-1α transcriptionally regulated protein CA-IX in normoxia, hypoxia and Dfx for T24, 5637 and HT1376 cell lines. The graph shows a statistically significant increase in the expression of CA-IX under hypoxia and Dfx in comparison to normoxia. Graphs represent average value of three independent experiments, $*p < 0.05$; $**p < 0.01$ (Student's *T*-test).

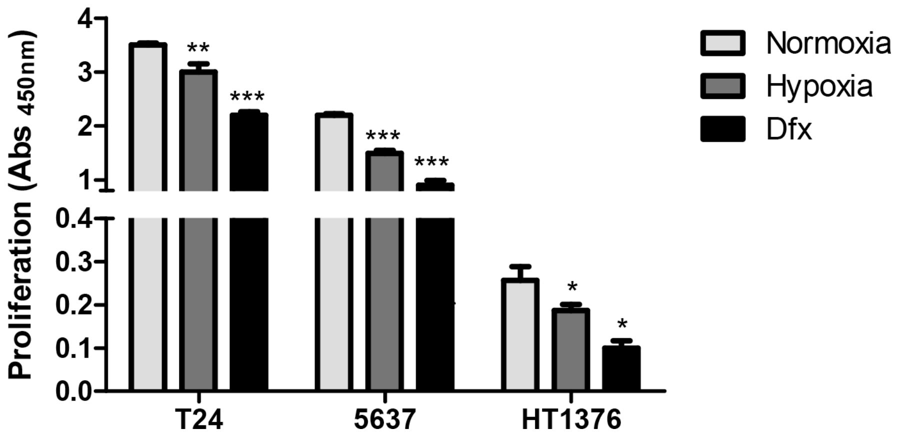

Supplementary Figure S2: Cell proliferation in normoxia, hypoxia and Dfx for T24, 5637 and HT1376 cell lines. The graph shows a statistically significant reduction in cell proliferation in hypoxia and Dfx in comparison to normoxia in all cell lines. It also shows that HT1376 cell line is less proliferative than T24 and 5637, irrespectively of the condition. Graphs represent average value of three independent experiments. $*p < 0.05$; $**p < 0.01$; $***p < 0.001$ (Student's *T*-test).

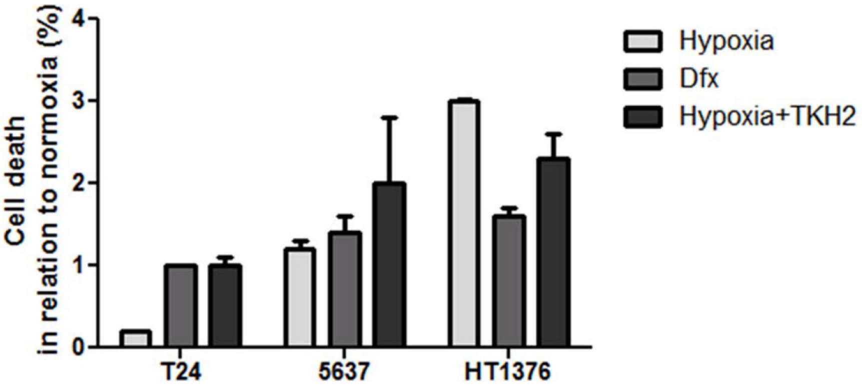

Supplementary Figure S3: Percentage of cell death in relation to normoxia for T24, 5637 and HT1376 under hypoxia, Dfx and Hypoxia in the presence of anti-STn monoclonal antibody TKH2. The results shows that growth in hypoxia, Dfx and exposure to the anti-STn antibody has minimum impact (> 5%) on cell viability, irrespectively of the cell line. Graphs represent average value of three independent experiments. *$p < 0.05$; **$p < 0.01$ (Student's *T*-test).

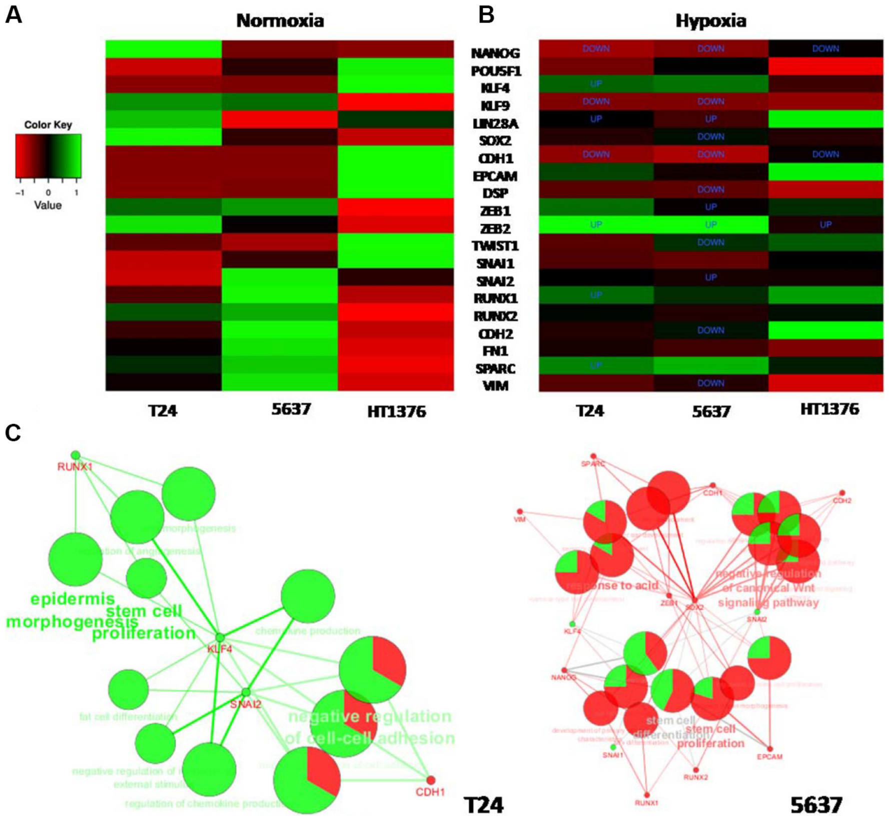

Supplementary Figure S4: (**A**) Heat map comparing the expression of genes associated with stem cells *(NANOG, LIN28A, POU5F1, KLF9, KLF4, SOX2)*; epithelial *(CDH1, DSP, EPCAM)*; Epithelial-to-mesenchymal transition *(SNAI1, SNAI2, TWIST1, TWIST2, ZEB1, ZEB2, RUNX1, RUNX2, FN1)*; mesenchymal *(CDH2, VIM, SPARC)* phenotypes in T24, 5637 and HT1376 cell lines. T24 and 5637 evidence a more pronounced stem and mesenchymal character when compared to HT1376. Note the lower expression of epithelial markers (CDH1, DSP, EPCAM) in T24 and 5637 and higher expression of these markers in HT1376 cells. Moreover, T24 present a more marked stem cell character while 5637 showed a more mesenchymal phenotype. (**B**) Heat map showing alterations in gene expression for each cell line under hypoxia in relation to normoxia. Statistically significant down an upregulations for each cell line are pointed out. Accordingly, we observed a higher number of alterations in 5637 cell line, while HT1376 was mostly unchanged in hypoxia compared to normoxia in relation to the targeted genes. (**C**) Biological interpretation of gene transcription alteration in hypoxia using ClueGO and CluePedia for Cystoscape. T24 gene expression profiles highlighted a positive regulation of stem cell proliferation, reinforcing the stem cell character of these cells, accompanied by the negative regulation of cell adhesion under hypoxia, thus in agreement with the morphological analysis. In 5637 cells, deregulations in stem cell proliferation as well as the activation of the Wnt signalling pathway, which has been associated with invasive and metastatic potential of cells, were amongst the most relevant alterations. The few alterations presented by HT1376 cells under hypoxia did not allow this type of analysis. Altogether, these observations showed that T24 and 5637 bladder cancer cells, presenting a more pronounced stem and mesenchymal character, were more affected by hypoxia, endowing them with a more aggressive phenotype compared to HT1376, which showed a more marked epithelial nature.

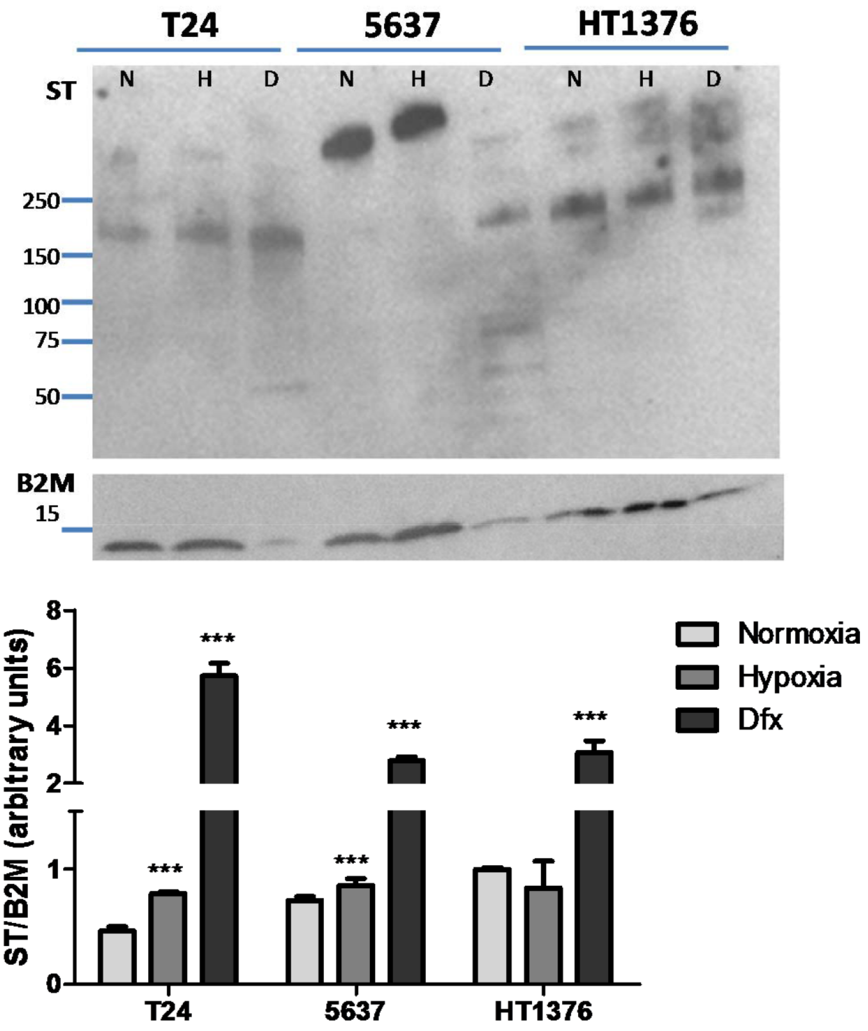

Supplementary Figure S5: Expression of the ST antigen in T24, 5637 and HT1376 cells in normoxia, hypoxia and exposure to Dfx. The ST antigen was determined after neuraminidase treament with the 3C9 monoclonal antibody against the T antigen, which is not expressed by these cell lines. Accordingly, hypoxia promotes a significant increase in ST expression in T24 and 5637 cell lines, but not in HT1376. Exposure to Dfx promotes an increase in this antigen in all cases, denoting a potential involvment of HIF-1α in ST regulation.

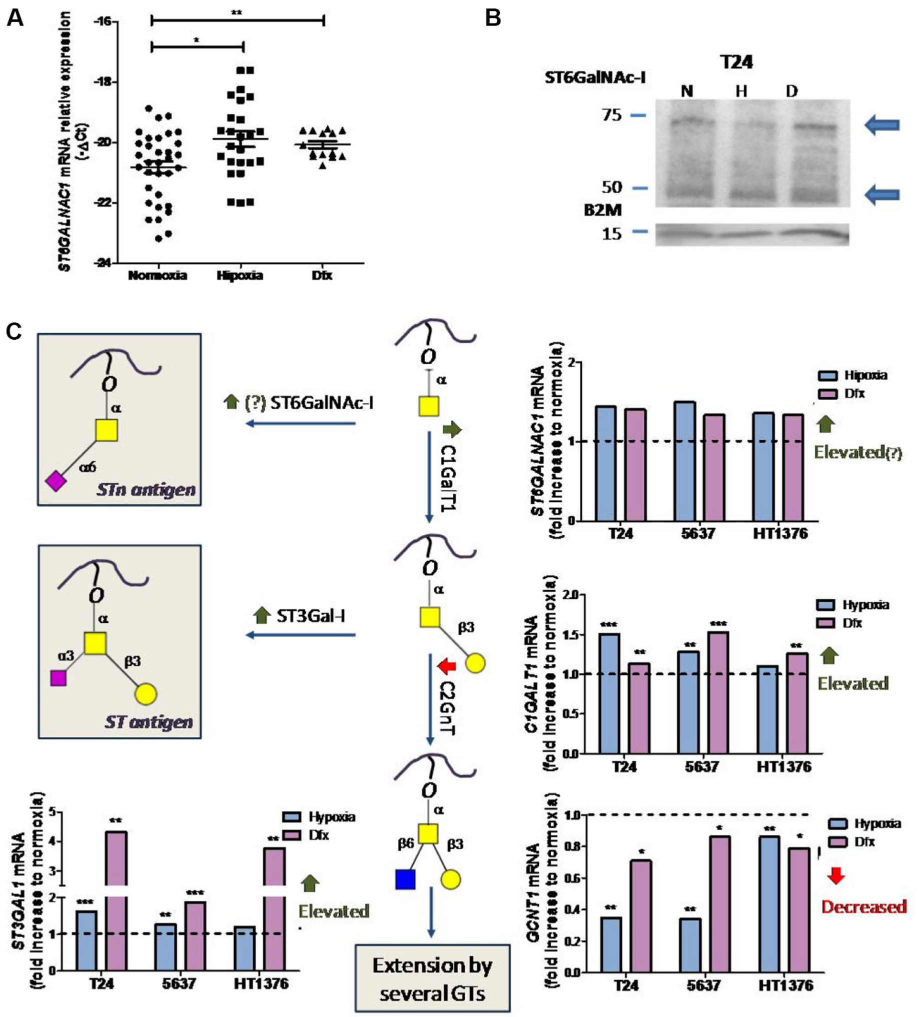

Supplementary Figure S6: (**A**) Variations in *ST6GALNAC1* transcription (average expression: 3 transcripts/10^6 reference genes) in hypoxia and Dfx compared to normoxia. *ST6GALNAC1* transcription was increased in all cell lines and experimental conditions (hypoxia and Dfx) when compared to normoxia (upper graph). These observations were significantly more pronounced when considering all cell lines (lower graph). Data further suggests that *ST6GALNAC1* transcription is regulated by HIF-1α. (**B**) Exemplificative western blot for T24 cell line showing equal amounts of ST6GalNAC-I in normoxia, hypoxia and Dfx. Given the low levels of *ST6GALNAC1* transcripts, western blots were performed to confirm its presence. Accordingly, two main bands could be observed, below 75 kDa and near 50 kDa (blue arrows), corresponding to two different splice variants of this protein that have been described as fully functional. Similar results were obtained for the other two cell lines (data not shown). Based on these results, it was not possible to confirm the increase in ST6GalNAc-I suggested by RT-PCR. (**C**) Schematic representation of the initial steps of *O*-glycosylation, with emphasis on the mRNA overexpression of genes encoding glycosyltransferases (*ST6GALNAC1, C1GALT1, GCNT1* and *ST3GAL1*) during hypoxia and Dfx treatments in relation to normoxia. Accordingly, we observed a trend increased in *ST6GALNAC1* gene expression, encoding ST6GalNAc-I (responsible by the formation of the STn antigen; average expression: 3 transcripts/10^6 reference genes) in hypoxia and Dfx in relation to normoxia. These observations may, in part, account for the increase in STn expression in these conditions. In addition, we have observed a statistically significant increase in *C1GALT1* transcription (average expression: 3 transcripts/10^2 reference genes) accompanied by a decrease in *GCNT1* (average expression: 2 transcripts/10^2 reference gene) that encodes for C2GnT, responsible by further elongation in O-glycans. This was particularly notorious for T24 and 5637 cell lines (> 60%) in comparison to HT1376 (approximately 20%). Moreover, the significant overexpression of *ST3GAL1* (average expression: 1 transcript/10^2 reference gene) may decisively contribute to an overexpression of the ST antigen. Altogether, these findings point towards an accumulation of short-chained sialylated O-glycans and inhibition of further O-glycan extension in hypoxia. Similar observations were made in cells exposed to Dfx treatment, suggesting that glycosyltransferase transcription is regulated by HIF-1α. Noteworthy, these events were observed in all cell lines, highlighting common responses regarding the transcriptional regulation of glycosyltransferases. These findings warrant deeper investigation in future studies. All graphs in this figure represent average value of three independent experiments, *$p < 0.05$; **$p < 0.01$; ***$p < 0.001$ (Student's *T*-test).

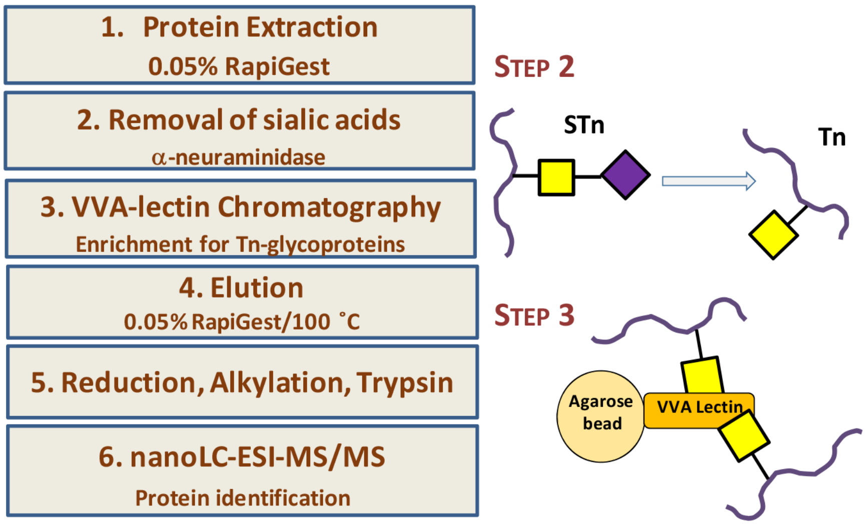

Supplementary Figure S7: Schematic representation of the STn-expressing glycoproteins isolation and identification protocol. Briefly proteins were extracted from normoxic and hypoxic cells with 0.05% RapiGest, whose amphiphilic nature allows a good representation of membrane proteins, which commonly yield *O*-GalNac glycosylation (Step 1). The extracts were then treated with an α-neuraminidase, which removes the sialic acid from STn exposing the Tn antigen (Step 2). The extracts were then enriched for Tn-expressing glycoproteins by *Vicia villosa* agglutinin (VVA)-lectin affinity chromatography (Step 3) and eluted with 0.05% RapiGest at 100°C after extensive wash of unbound material (Step 4). The isolated glycoproteins were then reduced, alkylated and digested with trypsin (Step 5) and identified by nanoLC-ESI-MS/MS (Step 6). Since Tn antigen was not detected by flow cytometry and western blot in these cell lines we assume that its expression is negletabe and that the glycoproteins identified by this approach mainly correspond to STn-expressing glycoproteins.

A **MUC16**

T24 **HYPOXIA**

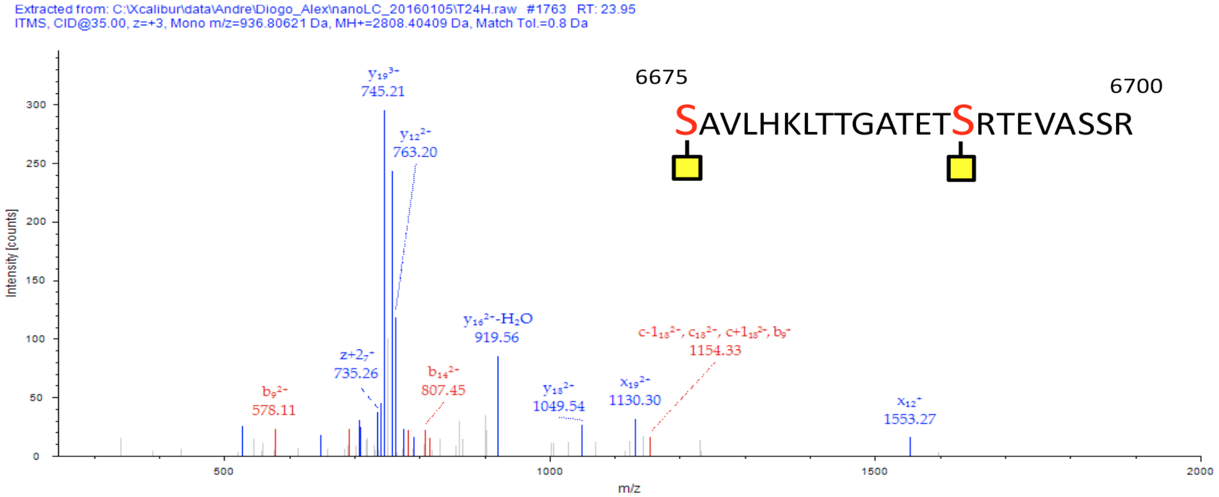

#1	a-(HexNAc+H_2O)$^{2+}$	a-H_2O^{3+}	a-NH_3^{3+}	b-(HexNAc+H_2O)$^{+}$	b-H_2O^{3+}	b-NH_3^{3+}	Seq.	y-H_2O^{2+}	y-(HexNAc+H_2O)$^{2+}$	y-H_2O^{3+}	y-NH_3^{2+}	y-NH_3^{3+}	#2
1							S-HexNAc						23
2							A						22
3							V						21
4							L						20
5							H			739.71		740.04	19
6							K						18
7				731.47			L						17
8							T	919.94					16
9							T		717.36				15
10							G						14
11							A			527.26	790.87	527.58	13
12							T						12
13							E						11
14							T						10
15							S-HexNAc						9
16							R						8
17	854.98						T						7
18	919.50						E						6
19		781.74	782.07		791.07	791.39	V						5
20	1004.55				814.75	815.08	A						4
21							S						3
22							S						2
23							R						1

B **INTEGRIN BETA-2**

HT1376 HYPOXIA

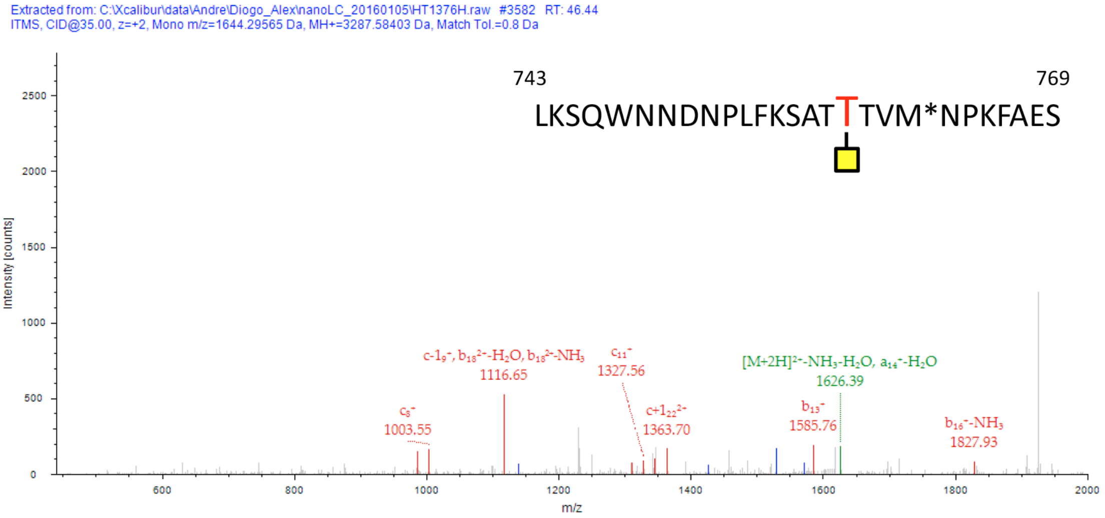

#1	a-HexNAc²⁺	b⁺	b-HexNAc²⁺	c⁺	c²⁺	c-HexNAc²⁺	c+1⁺	c+1²⁺	Seq.	x⁺	x-HexNAc⁺	x²⁺	y-HexNAc+	y-HexNAc²⁺	z-HexNAc²⁺	#2
1									L							27
2									K							26
3									S			1435.16				25
4									Q			1391.64				24
5									W			1327.61		1314.62		23
6									N							22
7									N			1177.55				21
8		986.47		1003.49					D							20
9				1117.54					N					1050.03		19
10									P							18
11		1310.65		1327.68			1328.68		L		1800.85					17
12									F						1760.87	16
13		1585.81							K					814.40	1613.80	15
14									S					750.36	1485.71	14
15									A				1412.68			13
16									T	1570.69	1266.58					12
17									T-HexNAc				1240.59			11
18	1010.02					1032.54			T							10
19									V							9
20									M-Oxidation							8
21									N							7
22								1363.17	P							6
23	1302.67		1316.67		1426.72	1325.18		1427.22	K							5
24									F							4
25									A							3
26									E							2
27									S							1

C PROTOCADHERIN 23

T24 HYPOXIA

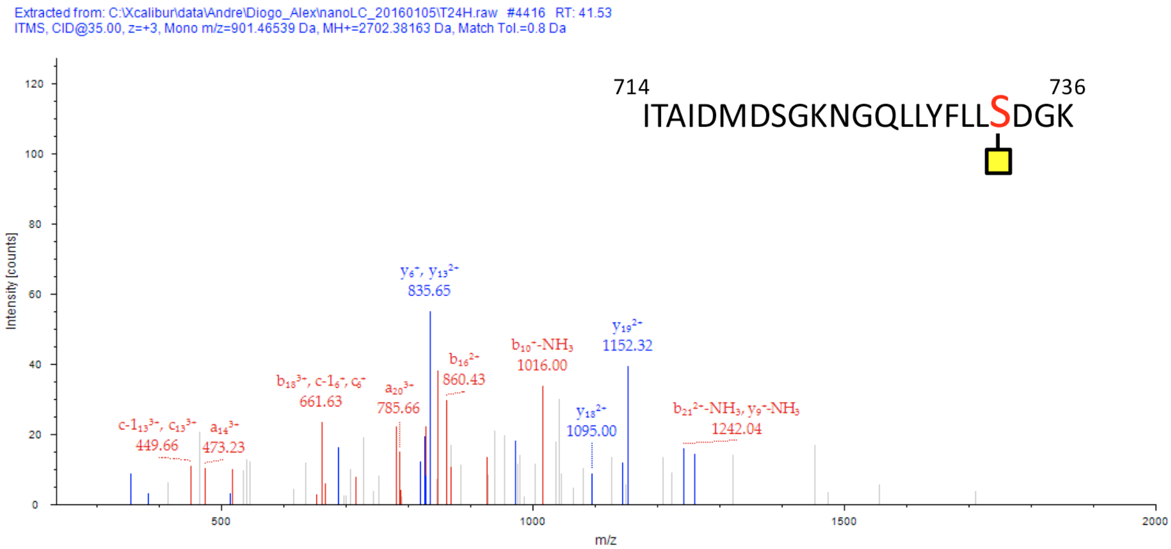

14	a^+	a^{2+}	a^{3+}	b^+	b^{2+}	b^{3+}	b-HexNAc^{3+}	c-1$^+$	c-1^{2+}	c-1^{3+}	c^+	c^{2+}	c^{3+}	Seq.	y^+	y^{2+}	y-HexNAc^{2+}	y^{3+}	#2
1														I					23
2														T					22
3														A					21
4														I					20
5				514.28										D		1152.55	927.98		19
6								661.34			662.35			M		1095.04			18
7														D			826.95	686.68	17
8	819.39			847.38										S		972.00			16
9														G					15
10					516.75									K					14
11						382.85								N		835.93			13
12														G					12
13		652.31			666.31					449.88			450.22	Q					11
14			472.90											L		686.37			10
15									787.41					L	1258.65				9
16		846.93			860.93				868.94					Y				382.52	8
17														F					7
18						661.00				666.34			666.68	L	835.44				6
19														L					5
20			786.75				727.71							S-HexNAc					4
21									1258.12			1258.63		D					3
22														G					2
23														K					1

Supplementary Figure S8: Examples of annotated nanoLC-ESI-MS^2 Tn-glycopeptides spectra for (A) MUC16; (B) Integrin beta-2; (C) Protocadherin-23. A table presenting key ions for assigment of the peptide and glycosylation sites is presented bellow each spectrum.

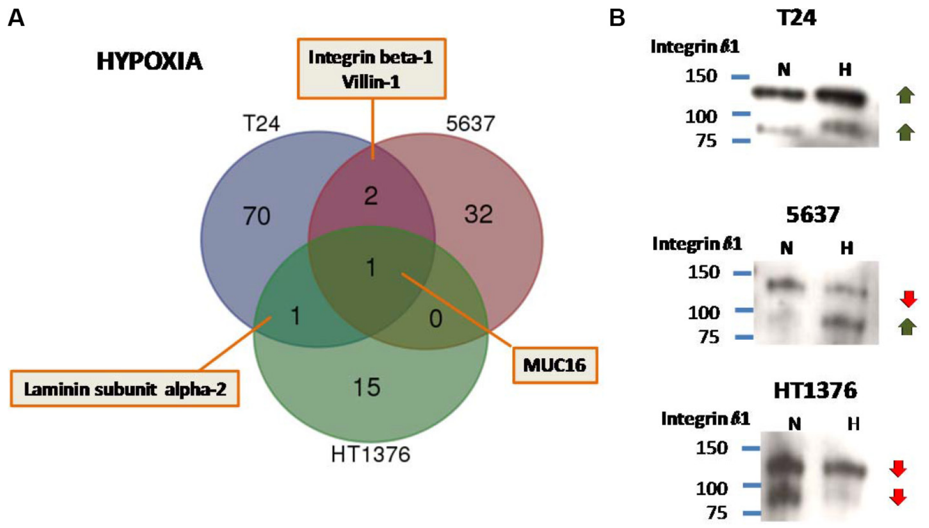

Supplementary Figure S9: (**A**) Distribution of putative STn-expressing glycoproteins between the three cell lines in hypoxia. The Venn diagram reinforces the notion that MUC16 is present in all cell lines in hypoxia. Interestingly, T24 and 5637 also shared the expression of integrin beta-1, which plays a critical role in cell adhesion and invasion and was previously described to be modified with the STn antigen [42–44]. (**B**) Expression of integrin beta-1 isolated by VVA lectin-affinity chromatography after neuraminidase treatment. Briefly, proteins isolated from T24, 5637 and HT1376 cells in normoxia and hypoxia were digested with α-neuraminidase, which removes the sialic acid from STn exposing the Tn antigen. The samples were then enriched for Tn-expressing glycoproteins by VVA affinity chromatography normalized in relation to the amount of used lectin. The glycoproteins were then separated by SDS-PAGE and blotted for integrin beta-1. Two bands could be observed (at approximately 100 and 150 kDa) in all cell lines and conditions, corresponding to the two main glycoforms of the protein. In agreement with this observation, integrin beta-1 has 13 putative O-glycosylation sites (according to NetOGlyc 4.0) and 12 putative N-glycosylations sites (according to NetNGlyc 1.0), that may account for these differences. Furthermore, these observations led to the conclusion that altered glycosylation is present in high as well as in low molecular weight integrin beta-1 glycoforms. Interestingly, western blot allowed the detection of integrin beta-1 in normoxia in all cell lines and in hypoxic HT1376, which were not identified by MS. The Figure also shows that hypoxia led to an overall increase in integrin beta-1 in T24 and 5637 cells, which would explain their identification by MS. Noteworthy, both cell lines presented a significant increase in low molecular weight integrin beta-1 glycoforms in hypoxia, which probably yields a higher number of short-chain O-glycans. Contrastingly, hypoxic HT1376 cells expressed significantly lower levels of both glycoforms in hypoxia when compared to normoxia. These observations support the notion that the STn antigen may be present in both low and high molecular weight integrin beta-1 glycoforms. Furthermore, it highlights that changes in the O-glycome accompanying hypoxia may result from both alterations in glycosylation pathways and the proteome. Future studies should devote to understanding the biological implications of these alterations.

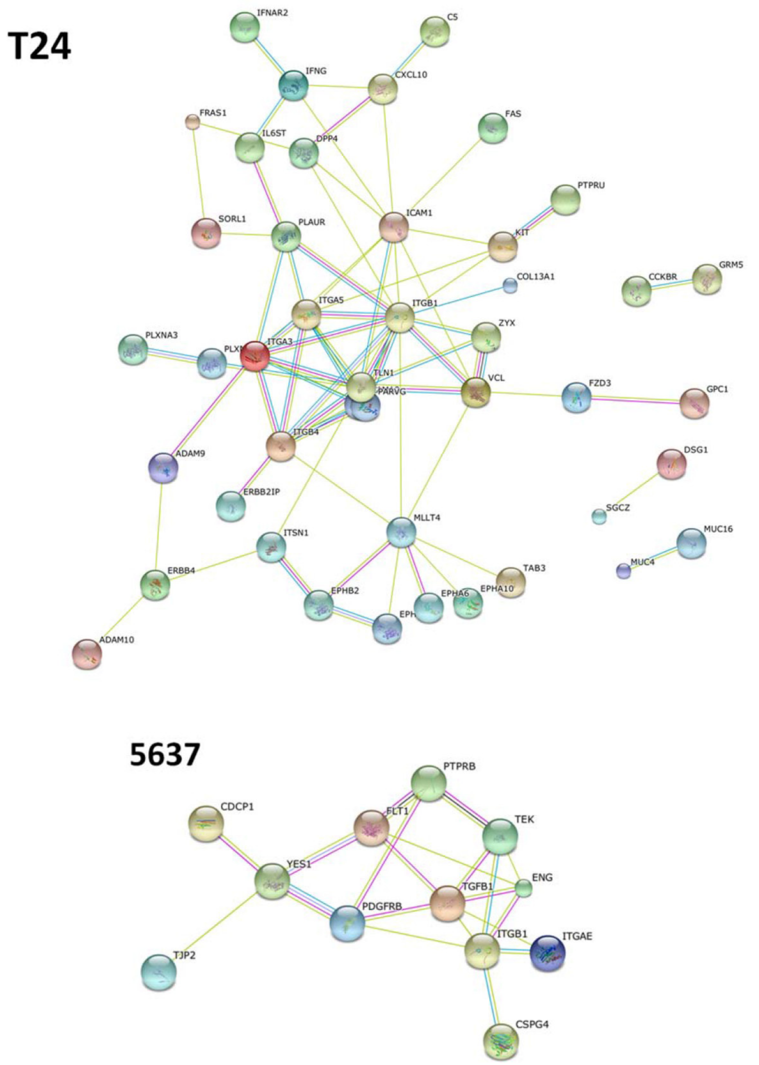

Supplementary Figure S10: Protein-Protein interactions given by STRING analysis for putative STn-expressing glycoproteins in hypoxia for (disconnected nods have been removed). Integrin beta-1 is a hub protein in terms of cell adhesion related interactions in both T24 and 5637 cell lines, further reinforcing the need for deeper investigations regarding its biological and clinical significance in bladder cancer. Importantly, the integration of HT1376 glycoproteins did not retrieve any functional relationships.

Supplementary Table S1: T24 cell line membrane glycoproteins putatively substituted with the STn antigen identified by VVA lectin affinity chromatography nanoLC-ESI-MS/MS. See Supplementary Table_S1

Supplementary Table S2: 5637 cell line membrane glycoproteins putatively substituted with the STn antigen identified by VVA lectin affinity chromatography nanoLC-ESI-MS/MS. See Supplementary Table_S2

Supplementary Table S3: HT1376 cell line membrane glycoproteins putatively substituted with the STn antigen identified by VVA lectin affinity chromatography nanoLC-ESI-MS/MS

Accession	Description	Coverage	MW [kDa]
	**HT1376 Normoxia**		
O60503	Adenylate cyclase type 9	1.63	150.6
P0C0L4	Complement C4-A	1.72	192.7
Q9UMR7	C-type lectin domain family 4 member A	11.39	27.5
Q9H2U9	Disintegrin and metalloproteinase domain-containing protein 7	3.98	85.6
P36269	Gamma-glutamyltransferase 5	2.73	62.2
Q9BX51	Gamma-glutamyltransferase light chain 1	17.78	24.3
P30462	HLA class I histocompatibility antigen, B-14 alpha chain	5.52	40.3
Q14573	Inositol 1,4,5-trisphosphate receptor type 3	0.79	303.9
Q8WXI7	Mucin-16	0.56	235.1
Q9NZM1	Myoferlin	0.78	234.6
P35579	Myosin-9	0.71	226.4
Q12913	Receptor-type tyrosine-protein phosphatase eta	1.05	145.9
Q6EMK4	Vasorin	5.20	71.7
O15231	Zinc finger protein 185	3.92	73.5
	**HT1376 Hypoxia**		
O60241	Adhesion G protein-coupled receptor B2	2.15	172.5
O00192	Armadillo repeat protein deleted in velo-cardio-facial syndrome	1.77	104.6
Q04656	Copper-transporting ATPase 1	2.40	163.3
Q9UJU6	Drebrin-like protein	2.33	48.2
Q04609	Glutamate carboxypeptidase 2	3.60	84.3
P05107	Integrin beta-2	3.51	84.7
Q86YT9	Junctional adhesion molecule-like	4.06	44.3
P24043	Laminin subunit alpha-2	1.09	343.7
Q96QZ7	Membrane-associated guanylate kinase, WW and PDZ domain-containing protein 1	0.87	164.5
Q8WXI7	Mucin-16	0.57	235.1
P08183	Multidrug resistance protein 1	2.42	141.4
O15439	Multidrug resistance-associated protein 4	2.34	149.4
P20138	Myeloid cell surface antigen CD33	9.62	39.8
Q15124	Phosphoglucomutase-like protein 5	3.17	62.2
Q9ULI3	Protein HEG homolog 1	1.67	147.4
Q9HC56	Protocadherin-9	1.62	136.0
O60603	Toll-like receptor 2	2.68	89.8
Q12866	Tyrosine-protein kinase Mer	2.60	110.2

Supplementary Table S4: TaqMan gene expression assays references used to assess transcript levels for the genes of interest ordered according to molecular and cellular function

Cell Function	Gene	TaqMan Assay reference
**Self-renewal and stemness**		
	NANOG	Hs04260366_g1
	LIN28A	Hs01552405_g1
	POU5F1 (OCT-4)	Hs04260367_g1
	KLF9	Hs00230918_m1
	KLF4	Hs00358837_g1
	SOX2	Hs01053049_s1
**Epithelial phenotype**		
	CDH1	Hs01023894_m1
	DSP	Hs00950591_m1
	EpCAM	Hs00901885_m1
**Epithelial-to-mesenchymal transition (EMT)**		
	SNAI1	Hs00195591_m1
	SNAI2	Hs00950344_m1
	TWIST1	Hs01675818_s1
	TWIST2	Hs00382379_m1
	ZEB1	Hs01566410_m1
	ZEB2	Hs00207691_m1
	RUNX1	Hs01021971_m1
	RUNX2	Hs01047973_m1
	FN1	Hs00365052_m1
**Mesenchymal phenotype**		
	CDH2	Hs00983056_m1
	VIM	Hs00185584_m1
	SPARC	Hs00234160_m1
**Glycosyltransferases**		
	ST6GALNAC1	Hs00300842_m1
	C1GALT1	
	GCNT1	
	ST3GAL1	
**Reference Genes**		
	B2M	Hs00984230_m1
	HPRT	Hs99999909_m1
