# Supplementary material for: Hypoxia enhances the malignant nature of bladder cancer cells and concomitantly antagonizes protein O-glycosylation extension
Source: Oncotarget. 2016 Aug 12;7(39):63138–57. doi: 10.18632/oncotarget.11257 (PMC5325352; doi:10.18632/oncotarget.11257)
Supplement: Supplementary file 2 [file oncotarget-07-63138-s002.docx]

**Table S1.** T24 cell line membrane glycoproteins putatively substituted with the STn antigen identified by VVA lectin affinity chromatography nanoLC-ESI-MS/MS

| **Accession** | **Description** | **Coverage (%)** | **MW [kDa]** | **O-Glycosylation sites** |
| --- | --- | --- | --- | --- |
|  |  |  |  |  |
| **T24 Normoxia** | | | | |
|  |  |  |  |  |
| Q8IZF2 | Adhesion G protein-coupled receptor F5 | 1.04 | 149.4 |  |
| P08F94 | Fibrocystin | 0.61 | 446.4 |  |
| P02751 | Fibronectin | 1.01 | 262.5 |  |
| O76083 | High affinity cGMP-specific 3',5'-cyclic phosphodiesterase 9A | 3.37 | 68.4 |  |
| P01871 | Ig mu chain C region | 7.30 | 49.3 |  |
| Q13797 | Integrin alpha-9 | 2.51 | 114.4 |  |
| Q13255 | Metabotropic glutamate receptor 1 | 0.84 | 132.3 |  |
| Q8WXI7 | Mucin-16 | 0.13 | 2351.2 |  |
| Q9ULB1 | Neurexin-1 | 2.44 | 161.8 |  |
| Q9UHC9 | Niemann-Pick C1-like protein 1 | 0.88 | 148.6 |  |
| Q9UIW2 | Plexin-A1 | 0.63 | 210.9 |  |
| P56705 | Protein Wnt-4 | 4.56 | 39.0 |  |
| Q13018 | Secretory phospholipase A2 receptor | 0.96 | 168.5 |  |
| Q9UPR5 | Sodium/calcium exchanger 2 | 1.41 | 100.3 |  |
| Q9BX84 | Transient receptor potential cation channel subfamily M 6 | 1.58 | 231.6 |  |
| P32019 | Type II inositol 1,4,5-trisphosphate 5-phosphatase | 1.51 | 112.8 |  |
| Q01668 | Voltage-dependent L-type calcium channel subunit alpha-1D | 0.74 | 245.0 |  |
|  |  |  |  |  |
| **T24 Hypoxia** | | | | |
|  |  |  |  |  |
| P55196 | Afadin | 1.32 | 206.7 |  |
| Q9HCM4 | Band 4.1-like protein 5 | 8.73 | 81.8 |  |
| Q9ULB5 | Cadherin-7 | 2.80 | 87.0 |  |
| Q5TAT6 | Collagen alpha-1(XIII) chain | 3.63 | 69.9 |  |
| Q96A83 | Collagen alpha-1(XXVI) chain | 7.26 | 45.4 |  |
| P01031 | Complement C5 | 2.27 | 188.2 |  |
| P02778 | C-X-C motif chemokine 10 | 18.37 | 10.9 |  |
| O95727 | Cytotoxic and regulatory T-cell molecule | 4.07 | 44.6 |  |
| Q02413 | Desmoglein-1 | 9.44 | 113.7 |  |
| P27487 | Dipeptidyl peptidase 4 | 3.00 | 88.2 |  |
| O14672 | Disintegrin and metalloproteinase domain-containing protein 10 | 2.14 | 84.1 |  |
| Q13443 | Disintegrin and metalloproteinase domain-containing protein 9 | 1.59 | 90.5 |  |
| Q68DV7 | E3 ubiquitin-protein ligase RNF43 | 1.53 | 85.7 |  |
| Q16206 | Ecto-NOX disulfide-thiol exchanger 2 | 7.05 | 70.0 |  |
| Q9NZN3 | EH domain-containing protein 3 | 3.36 | 60.8 |  |
| Q5JZY3 | Ephrin type-A receptor 10 | 2.58 | 109.6 |  |
| Q9UF33 | Ephrin type-A receptor 6 | 0.97 | 116.3 |  |
| P54762 | Ephrin type-B receptor 1 | 2.54 | 109.8 |  |
| P29323 | Ephrin type-B receptor 2 | 2.84 | 117.4 |  |
| Q86XX4 | Extracellular matrix protein FRAS1 | 0.70 | 442.9 |  |
| Q68DX3 | FERM and PDZ domain-containing protein 2 | 2.75 | 144.2 |  |
| Q9NPG1 | Frizzled-3 | 4.05 | 76.2 |  |
| Q6ZNL6 | FYVE, RhoGEF and PH domain-containing protein 5 | 2.46 | 159.8 |  |
| Q9HBI0 | Gamma-parvin | 4.83 | 37.5 |  |
| P32239 | Gastrin/cholecystokinin type B receptor | 5.82 | 48.4 |  |
| P35052 | Glypican-1 | 3.05 | 61.6 |  |
| Q9NZH0 | G-protein coupled receptor family C group 5 member B | 4.71 | 44.8 |  |
| O60478 | Integral membrane protein GPR137B | 8.02 | 45.6 |  |
| P26006 | Integrin alpha-3 | 3.14 | 116.5 |  |
| P08648 | Integrin alpha-5 | 2.00 | 114.5 |  |
| P05556 | Integrin beta-1 | 5.01 | 88.4 |  |
| P16144 | Integrin beta-4 | 0.66 | 202.0 |  |
| P05362 | Intercellular adhesion molecule 1 | 3.76 | 57.8 |  |
| P48551 | Interferon alpha/beta receptor 2 | 2.14 | 57.7 |  |
| P01579 | Interferon gamma | 7.23 | 19.3 |  |
| P40189 | Interleukin-6 receptor subunit beta | 2.40 | 103.5 |  |
| Q15811 | Intersectin-1 | 0.58 | 195.3 |  |
| P24043 | Laminin subunit alpha-2 | 0.86 | 343.7 |  |
| Q86UK5 | Limbin | 2.14 | 147.9 |  |
| P10721 | Mast/stem cell growth factor receptor Kit | 2.56 | 109.8 |  |
| P41594 | Metabotropic glutamate receptor 5 | 1.40 | 132.4 |  |
| Q29980 | MHC class I polypeptide-related sequence B | 2.61 | 42.6 |  |
| Q8WXI7 | Mucin-16 | 0.47 | 2351.2 |  |
| Q99102 | Mucin-4 | 1.34 | 231.4 |  |
| Q9UIW2 | Plexin-A1 | 1.85 | 210.9 |  |
| P51805 | Plexin-A3 | 1.60 | 207.6 |  |
| Q9Y4D7 | Plexin-D1 | 0.99 | 211.9 |  |
| Q96RT1 | Protein LAP2 | 5.95 | 158.2 |  |
| Q04941 | Proteolipid protein 2 | 9.87 | 16.7 |  |
| Q14517 | Protocadherin Fat 1 | 0.33 | 506.0 |  |
| Q9NYQ8 | Protocadherin Fat 2 | 1.03 | 479.0 |  |
| Q08174 | Protocadherin-1 | 3.58 | 114.7 |  |
| Q8TAB3 | Protocadherin-19 | 2.09 | 126.2 |  |
| Q6V1P9 | Protocadherin-23 | 0.79 | 322.0 |  |
| P04201 | Proto-oncogene Mas | 3.69 | 37.4 |  |
| Q15303 | Receptor tyrosine-protein kinase erbB-4 | 2.14 | 146.7 |  |
| Q15262 | Receptor-type tyrosine-protein phosphatase kappa | 2.78 | 162.0 |  |
| Q13332 | Receptor-type tyrosine-protein phosphatase S | 0.98 | 216.9 |  |
| Q92729 | Receptor-type tyrosine-protein phosphatase U | 0.76 | 162.3 |  |
| P11166 | Solute carrier family 2, facilitated glucose transporter member 1 | 4.88 | 54.0 |  |
| Q92673 | Sortilin-related receptor | 0.63 | 248.3 |  |
| Q9Y490 | Talin-1 | 1.46 | 269.6 |  |
| Q9UKZ4 | Teneurin-1 | 0.59 | 304.8 |  |
| Q9NT68 | Teneurin-2 | 0.40 | 307.6 |  |
| Q8N5C8 | TGF-beta-activated kinase 1 and MAP3K7-binding protein 3 | 4.92 | 78.6 |  |
| Q9UPZ6 | Thrombospondin type-1 domain-containing protein 7A | 1.03 | 185.2 |  |
| O75509 | Tumor necrosis factor receptor superfamily member 21 | 3.21 | 71.8 |  |
| P36941 | Tumor necrosis factor receptor superfamily member 3 | 6.21 | 46.7 |  |
| P25445 | Tumor necrosis factor receptor superfamily member 6 | 5.67 | 37.7 |  |
| Q03405 | Urokinase plasminogen activator surface receptor | 9.25 | 37.0 |  |
| P09327 | Villin-1 | 2.78 | 92.6 |  |
| P18206 | Vinculin | 1.94 | 123.7 |  |
| Q96LD1 | Zeta-sarcoglycan | 14.05 | 32.9 |  |
| Q15942 | Zyxin | 3.32 | 61.2 |  |
|  |  |  |  |  |
