# Supplementary material for: Hypoxia enhances the malignant nature of bladder cancer cells and concomitantly antagonizes protein O-glycosylation extension
Source: Oncotarget. 2016 Aug 12;7(39):63138–57. doi: 10.18632/oncotarget.11257 (PMC5325352; doi:10.18632/oncotarget.11257)
Supplement: Supplementary file 3 [file oncotarget-07-63138-s003.docx]

**Table S2.** 5637 cell line membrane glycoproteins putatively substituted with the STn antigen identified by VVA lectin affinity chromatography nanoLC-ESI-MS/MS.

| **Accession** | **Description** | **Coverage** | **MW [kDa]** | **O-Glycosylation sites** |
| --- | --- | --- | --- | --- |
|  |  |  |  |  |
| **5637 Normoxia** | | | | |
|  |  |  |  |  |
| Q4VCS5 | Angiomotin | 2.95 | 118.0 |  |
| O75882 | Attractin | 2.52 | 158.4 |  |
| Q14574 | Desmocollin-3 | 2.57 | 99.9 |  |
| P52799 | Ephrin-B2 | 5.11 | 36.9 |  |
| Q05397 | Focal adhesion kinase 1 | 2.85 | 119.2 |  |
| Q96J84 | Kin of IRRE-like protein 1 | 2.11 | 83.5 |  |
| P48039 | Melatonin receptor type 1A | 5.14 | 39.3 |  |
| Q8WXI7 | Mucin-16 | 0,30 | 235.,1 |  |
| O14786 | Neuropilin-1 | 1.84 | 103.1 |  |
| Q8TF62 | Probable phospholipid-transporting ATPase IM | 1.01 | 135.8 |  |
| P23470 | Receptor-type tyrosine-protein phosphatase gamma | 0.76 | 161.9 |  |
| Q9P0X4 | Voltage-dependent T-type calcium channel subunit alpha-1I | 0.40 | 244.9 |  |
| Q9UBH6 | Xenotropic and polytropic retrovirus receptor 1 | 1.87 | 81.5 |  |
|  |  |  |  |  |
| **5637 Hypoxia** | | | | |
|  |  |  |  |  |
| P25054 | Adenomatous polyposis coli protein | 2.22 | 311.5 |  |
| O43865 | Adenosylhomocysteinase 2 | 4.91 | 58.9 |  |
| P51828 | Adenylate cyclase type 7 | 3.06 | 120.2 |  |
| Q969X2 | Alpha-N-acetylgalactosaminide alpha-2,6-sialyltransferase 6 | 7.21 | 38.0 |  |
| Q02763 | Angiopoietin-1 receptor | 2.67 | 125.7 |  |
| Q6YHK3 | CD109 antigen | 1.87 | 161.6 |  |
| Q4KMG0 | Cell adhesion molecule-related/down-regulated by oncogenes | 2.87 | 139.1 |  |
| Q6UVK1 | Chondroitin sulfate proteoglycan 4 | 1.34 | 250.4 |  |
| Q9UMR7 | C-type lectin domain family 4 member A | 11.39 | 27.5 |  |
| Q9H5V8 | CUB domain-containing protein 1 | 3.11 | 92.9 |  |
| Q6XUX3 | Dual serine/threonine and tyrosine protein kinase | 2.69 | 105.1 |  |
| Q8TC92 | Ecto-NOX disulfide-thiol exchanger 1 | 5.29 | 73.3 |  |
| P17813 | Endoglin | 5.93 | 70.5 |  |
| Q07075 | Glutamyl aminopeptidase | 3.13 | 109.2 |  |
| P30501 | HLA class I histocompatibility antigen, Cw-2 alpha chain | 4.64 | 41.1 |  |
| O75330 | Hyaluronan mediated motility receptor | 2.49 | 84.0 |  |
| P38570 | Integrin alpha-E | 2.63 | 130.1 |  |
| P05556 | Integrin beta-1 | 4.26 | 88.4 |  |
| Q86UP2 | Kinectin | 2.06 | 156.2 |  |
| Q8WXI7 | Mucin-16 | 0.76 | 2351.2 |  |
| Q6T4R5 | Nance-Horan syndrome protein | 1.76 | 179.0 |  |
| Q99650 | Oncostatin-M-specific receptor subunit beta | 2.86 | 110.4 |  |
| Q9H307 | Pinin | 3.35 | 81.6 |  |
| P09619 | Platelet-derived growth factor receptor beta | 1.81 | 123.9 |  |
| Q9Y4D7 | Plexin-D1 | 0.99 | 211.9 |  |
| Q96JQ0 | Protocadherin-16 | 0.61 | 346.0 |  |
| P23467 | Receptor-type tyrosine-protein phosphatase beta | 2.35 | 224.2 |  |
| P13866 | Sodium/glucose cotransporter 1 | 4.97 | 73.4 |  |
| Q9UHW9 | Solute carrier family 12 member 6 | 2.70 | 127.5 |  |
| Q9UDY2 | Tight junction protein ZO-2 | 3.03 | 133.9 |  |
| P01137 | Transforming growth factor beta-1 | 6.67 | 44.3 |  |
| P42680 | Tyrosine-protein kinase Tec | 2.69 | 73.5 |  |
| P07947 | Tyrosine-protein kinase Yes | 4.05 | 60.8 |  |
| P17948 | Vascular endothelial growth factor receptor 1 | 1.12 | 150.7 |  |
| P09327 | Villin-1 | 3.14 | 92.6 |  |
